# Discovery of novel potential selective HDAC8 inhibitors by combine ligand-based, structure-based virtual screening and *in-vitro* biological evaluation

**DOI:** 10.1038/s41598-019-53376-y

**Published:** 2019-11-20

**Authors:** Sudhan Debnath, Tanusree Debnath, Samhita Bhaumik, Swapan Majumdar, Arunasree M. Kalle, Vema Aparna

**Affiliations:** 1 Department of Chemistry, MBB College, Agartala, Tripura 799004 India; 2 Department of Chemistry, Women’s College, Agartala, Tripura 799001 India; 3 Department of Chemistry, Tripura University, Suryamaninagar, Agartala, Tripura 799022 India; 4 Department of Animal Biology, School of Life Sciences, University of Hyderabad, Hyderabad, TS 500046 India; 5 Sree Chaitanya Institute of Pharmaceutical Sciences, Karimnagar, 505 527 Andhra Pradesh India

**Keywords:** High-throughput screening, High-throughput screening, Structure-based drug design, Structure-based drug design

## Abstract

Neuroblastoma is the most common extracranial solid tumor found in children and survival rate is extremely meager. HDAC8, a class I zinc-dependent enzyme, is a potential drug target for treatment of neuroblastoma and T cell lymphoma. Most of the HDAC8 inhibitors discovered till date contains a hydroxamic acid group which acts as a zinc binding group. The high binding affinity to the zinc and other ions results in adverse effects. Also, the non-selective inhibition of HDACs cause a variety of side effects. The objective of this is to identify structurally diverse, non-hydroxamate, novel, potential and selective HDAC8 inhibitors. A number of five featured pharmacophore hypotheses were generated using 32 known selective HDAC8 inhibitors. The hypotheses ADDRR.4 were selected for building 3D QSAR model. This model has an excellent correlation coefficient and good predictive ability, which was employed for virtual screening of Phase database containing 4.3 × 10^6^ molecules. The resultant hits with fitness score >1.0 were optimized using in-silico ADMET (absorption, distribution, metabolism,  excretion, and toxicity) and XP glide docking studies. On the basis of pharmacophore matching, interacting amino acid residues, XP glide score, more affinity towards HDAC8 and less affinity towards other HDACs, and ADME results five hits- SD-01, SD-02, SD-03, SD-04 and SD-05 with new structural scaffolds,  non-hydroxamate were selected for *in vitro* activity study. SD-01 and SD-02 were found to be active in the nanomolar (nM) range. SD-01 had considerably good selectivity for HDAC8 over HDAC6 and SD-02 had marginal selectivity for HDAC6 over HDAC8. The compounds SD-01 and SD-02 were found to inhibit HDAC8 at concentrations (IC_50_) 9.0 nM and 2.7 nM, respectively.

## Introduction

HDACs are one of the most important classes of post-translational regulators that are responsible for deacetylation of lysine residues in histone and non-histone substrates. To date, 18 types of HDACs have been identified and classified into four broad classes: class I, II (further classified as IIa and IIb), III, and IV (Fig. S2). HDAC8, a class I zinc-dependent HDACs, which localizes to either the nucleus or the cytoplasm, typically induces histone deacetylation and represses gene transcription^1^. HDACs are identified as potential therapeutic targets due to their involvement in various diseases like cancer, inflammation, neurological disorders and infections^2,3^. In cancer, HDACs are either deregulated, over expressed, or interact with transcription factors^4^. HDAC8 may be the potential drug target for the treatment of minimal residual disease in neuroblastoma and malignancies such as T-cell lymphoma and acute myeloid leukemia^5,6^. FDA has approved suberoylanilide hydroxamic acid (vorinostat) for treatment of cutaneous T-cell lymphoma (CTCL) after multiple clinical trials in 2006^7,8^ and romidepsin (cyclic peptide) in 2009^9,10^. Many other HDAC inhibitors like belinostat^11^, panobinostat^12^, pracinostat^13^ have been approved by US-FDA and chidamide have been approved by China-FDA^14^ for treatment of cancer and several are in clinical trials^15^.

A number of structurally diverse HDAC8 inhibitors discovered so far are known to be hydroxamic acids^16–19^. Hydroxamic acid group binds with zinc, which often creates metabolic and pharmacokinetic troubles. Also, many hydroxamates are unstable *in vivo*, and on hydrolysis give mutagenic hydroxylamine^20^. Hydroxamic acid also showed a strong chelating ability with zinc and hence it lack selectivity^21^. Inhibition of several HDACs simultaneously confers greater toxicity and long term side effects. Therefore discovery of isoform-selective HDACs, improve therapeutic potential^22^. The highly conserved active site of HDACs family members makes it difficult to design isoform-selective inhibitors^19^. Thus, the discovery of potential, novel scaffolds and selective HDAC8 inhibitors besides currently existing hydroxamic acid is a necessity.

The combined ligand-based and structure-based approach is very important in modern drug discovery for searching potential lead molecules^23–27^. In the last few decades, the virtual screening tool was employed to identify novel lead molecules with diverse structural features^28–30^. The number of pharmacophore-based virtual screenings for identification of HDAC8 inhibitors against a commercial and in-house database of compounds has been reported to be small^31,32^ and the number of 3D QSAR model use for selective HDAC8 inhibitors has been reported to be very limited. This study aimed to find out novel non-hydroxamic acid, selective HDAC8 inhibitors using cost-effective and rapid in silico approach, a combination of pharmacophore-based virtual screening, molecular docking, ADMET (absorption, distribution, metabolism, excretion, and toxicity) properties and evaluation of *in vitro* HDAC8 and HDAC6 inhibitory activity of identified hits.

## Results and Discussions

**Figure 1:**
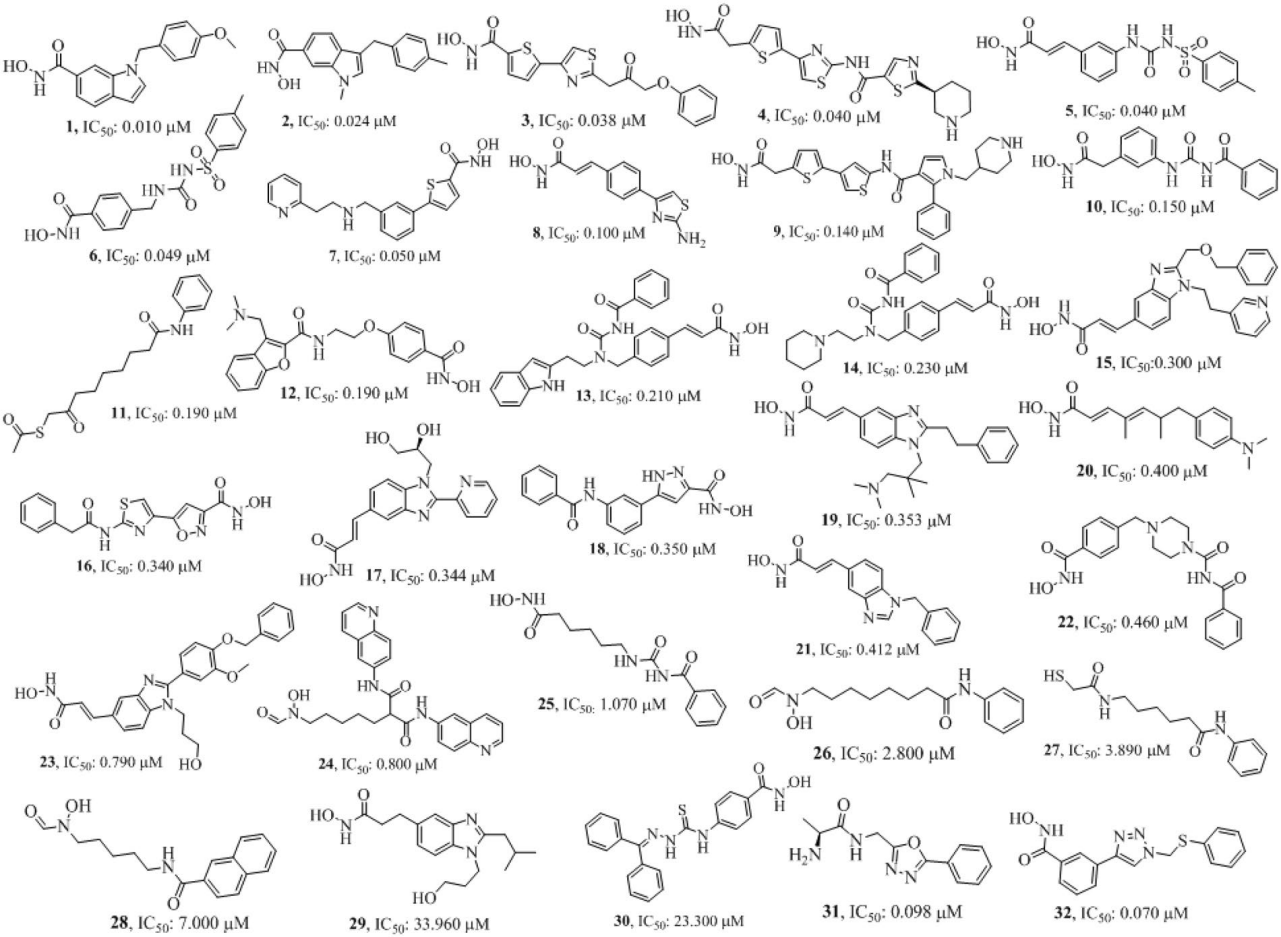
Structurally diverse 32 known selective HDAC8 inhibitors with their IC_50_ values used for 3D QSAR model building.

**Figure 2:**
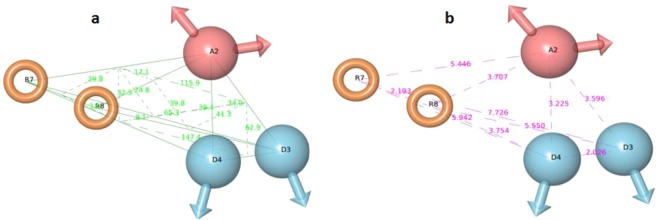
PHASE generated best pharmacophore model ADDRR.4 of selective HDAC8 inhibitors illustrating hydrogen bond acceptor (A2, pink), hydrogen bond donor (D3, D4; sky) and aromatic ring (R7, R8; orange) with their angles (**a**) are shown by green lines and distance (**b**) are shown purple lines. The twenty three training set inhibitors were used for this model generation.

**Figure 3:**
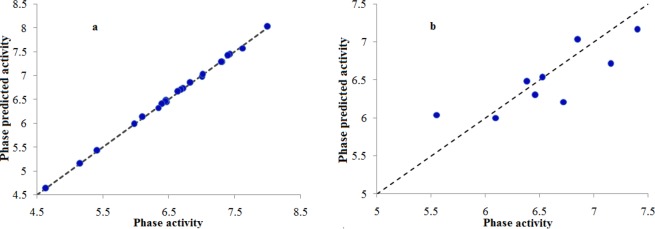
Fitness graph between observed activities of selective HDAC8 inhibitors and their PHASE activities predicted by generated by pharmacophore based 3D QSAR model of training set (**a**) and test set (**b**) of the inhibitors.

**Figure 4:**
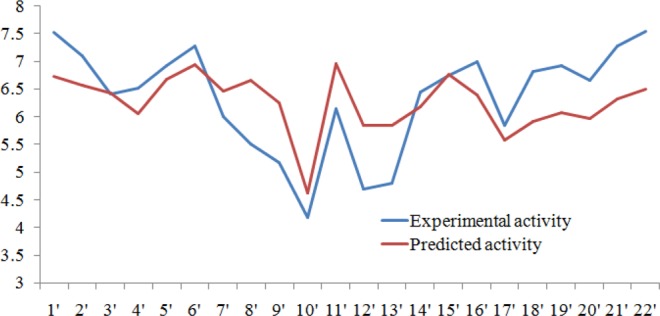
Experimental activity (EA) versus predicted activity (PA) of 22 known inhibitors used for validation of model.

The number of five-feature pharmacophore hypotheses were eleven, generated with a combination of three chemical features i.e. hydrogen-bond acceptor (A), hydrogen bond donor (D) and aromatic ring (R). The pharmacophore hypotheses ADDRR.4, AADRR.4, AADDR.12, AADDR.11, AADDR.15, AADDR.16, AADDR.14, AAADR.20, AAADR.19, AAADR.24, AAADR.23 were generated from 32 known selective HDAC8 inhibitors (Fig. 1). The 32 inhibitors were classified into eight series on the basis of their structural symmetry are series A–E, five and series F, which contains three diverse structures are shown in Fig. S1. Out of these pharmacophore hypotheses, three top-scoring hypotheses- ADDRR.4 (survival score: 5.139, survival-inactive: 3.298) AADRR.4 (survival score: 4.939, survival-inactive: 3.177), and AADDR.12 (survival score: 4.310, survival-inactive: 2.588) were selected for building 3D QSAR models. Out of three derived 3D QSAR models, ADDRR.4 was found to be statistically significant and the statistical parameters of the 3D QSAR model were R^2^ (squared correlation coefficient): 0.9995, with a root-mean-square error (RMSE) of 0.3023, the good F value of 8732.3. Its predictive correlation coefficient (Q^2^) which is 0.6626. (Table 1). The 3D QSAR model consisted of a spatial arrangement of five chemical features (Fig. 2). The predictive correlation coefficient confirmed the robustness and predictive ability of the model. The QSAR results showed that the observed activity of training and test set molecules were very close to phase predicted activity (Fig. 3 and Table S2). The predictive ability of the model has further been validated using known selective HDAC8 inhibitors 1’−22’ retrieved from the literature (Tables S1). The comparison of phase predicted pIC_50_ values of 22 external selective HDAC8 inhibitors from their corresponding experimental pIC_50_ (Fig. 4**)**, further supports that the predictive ability of the model was excellent. The best predictive 3D QSAR model built from AADRR.4 was used for virtual screening of Phase database. Pharmacophore-based virtual screening resulted in 500 hits fitness score >1.0 from Phase database.

**Table 1:** Statistical results of 3D-QSAR model generated from ADDRR.4.

ID	# Factors	SD	R-squared	F	P	Stability	RMSE	Q-squared	Pearson-R
ADDRR.4	1	0.3995	0.8077	92.4	2.47E-09	0.4886	0.298	0.6722	0.8292
	2	0.1494	0.9743	398.4	2.00E-17	0.2506	0.3169	0.6292	0.8191
	3	0.071	0.9945	1199.6	9.81E-23	0.2197	0.3084	0.6488	0.8235
	4	0.0229	0.9995	8732.3	1.02E-30	0.2019	0.3023	0.6626	0.8318

QickProp predicts physically significant and the pharmaceutically relevant properties of organic molecules by comparing 95% of known drugs. According to Lipinski’s rule of five for drug-like molecules the molecular weight should be <500, octanol-water partition coefficient (QPlogPo/w) should <5.0, hydrogen bond donor groups (donorHB) should be <5.0, and hydrogen bond acceptor groups should be ≤10. The predicted descriptors for all the five selected hits obeyed the Lipiniski’s rule of five. The QPlogPo/w and water solubility (QPlog S) which are essential in the evaluation of adsorption and distribution of drugs and the range of these parameters for five selected hits were 1.05–2.997 and −2.797–3.66, respectively, which were also in the acceptable range. The other important parameters are apparent Caco-2 cell permeability in nm/sec (QPPCaco) and apparent MDCK cell permeability in nm/sec (QPPMDCK) and the values were 47.626–973.33 and 43.612–2256.642, respectively. For all the five selected hits the values of QPPCaco and QPPMDCK are in the acceptable range but for potential HDAC8 inhibitor, these values for SD-01 were 973.33 and 2256.642, respectively which is great. The important ADME properties of finally identified five hits lie with acceptable range are listed in Table 2 and the remaining properties listed in Table S3.

**Table 2:** Prediction of ADMET properties of five hits.

Inhibitors	mol_MW	#stars	donorHB	accptHB	QPlogPo/w	QPlogS	QPPCaco
SD-01	385.498	0	3	6.25	2.997	−3.339	973.33
SD-02	306.318	0	3	6.25	1.597	−3.66	183.455
SD-03	369.33	0	2	8.25	1.05	−2.827	47.626
SD-04	292.739	0	2	4.50	1.847	−2.899	300.037
SD-05	260.292	0	2	4.45	1.879	−2.797	660.241
RV	130–725	0–5	0–6	2–20	−2–6.5	−6.5–0.5	<25 poor >500 great
Inhibitors	QPlogBB	QPPMDCK	PHOA	CNS	ROF	ROT	HOA
SD-01	−0.417	2256.642	100	−1	0	1	3
SD-02	−1.562	79.125	76.808	−2	0	0	3
SD-03	−1.977	43.612	63.124	−2	0	0	3
SD-04	−0.457	1307.828	82.095	0	0	0	3
SD-05	−0.609	632.067	88.413	0	0	0	3
RV	−3–1.2	<25 poor >500 great	>80% high <25% poor	−2–+2	Max. 4	Max. 3	3-high

RV: Recommended values; PHOA: Percent Human Oral Absorption; ROT: Rule Of Five; ROT: Rule Of Three; HOA: Human Oral Absorption.

**Figure 5:**
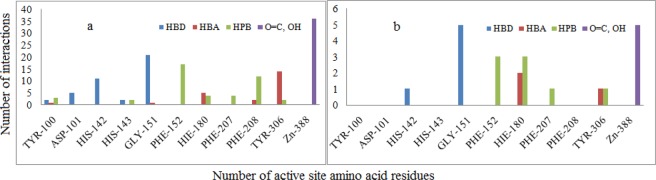
The number of different types of interactions of 32 known inhibitors (**a**) and 5 identified lead molecules (**b**) with different active site amino acid residues and Zn^+2^.

**Figure 6:**
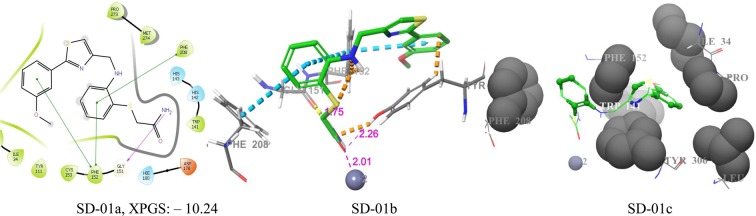
Docking poses of compound SD-01 ((**a**) 2D ligand interaction diagram like hydrogen bond donor, hydrogen bond acceptor, π-π stacking, (**b**) 3Dligandinteractions likehydrogen bond donor,hydrogen bond acceptor, π-π stacking, (**c**) 3D hydrophobic interactions). The 2D interactions are depicted with different colors: pi-pi (green line), hydrogen bond (violet line) for 3D interaction hydrogen bond (purple line), pi-pi (doted sky line).

**Figure 7:**
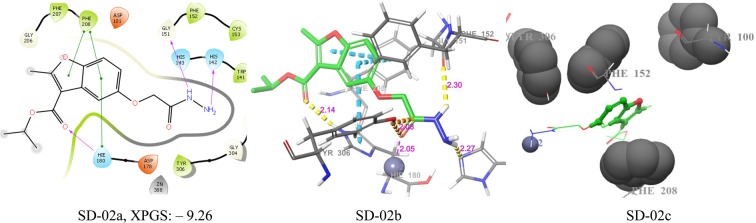
Docking poses of compound SD-02 ((**a**) 2D ligand interaction diagram like hydrogen bond donor, hydrogen bond acceptor, π-π stacking, (**b**) 3Dligandinteractions likehydrogen bond donor, hydrogen bond acceptor, π-π stacking, (**c**) 3D hydrophobic interactions). The 2D interactions are depicted with different colors: pi-pi (green line), hydrogen bond (violet line) for 3D interaction hydrogen bond (purple line), pi-pi (doted sky line).

**Figure 8:**
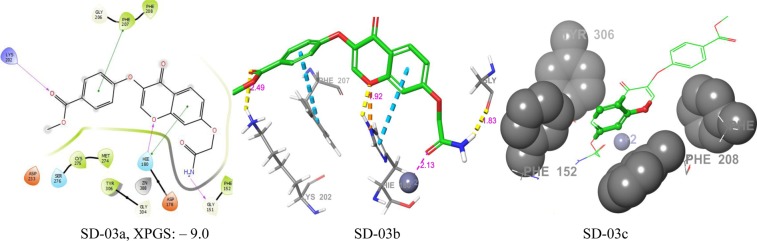
Docking poses of compound SD-03 ((**a**) 2D ligand interaction diagram like hydrogen bond donor, hydrogen bond acceptor, π-π stacking, (**b**) 3Dligandinteractions like hydrogen bond donor,hydrogen bond acceptor, π-π stacking, (**c**) 3D hydrophobic interactions). The 2D interactions are depicted with different colors: pi-pi (green line), hydrogen bond (violet line) for 3D interaction hydrogen bond (purple line), pi-pi (doted sky line).

**Figure 9:**
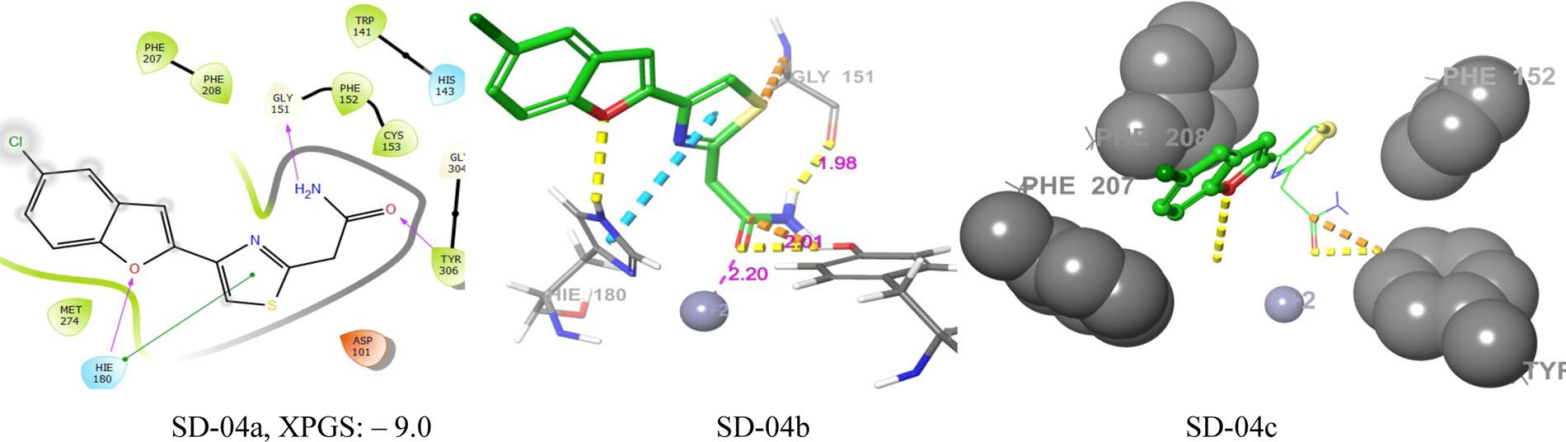
Docking poses of compound SD-04 ((**a**) 2D ligand interaction diagram like hydrogen bond donor, hydrogen bond acceptor, π-π stacking, (**b**) 3Dligandinteractions like hydrogen bond donor, hydrogen bond acceptor, π-π stacking, (**c**) 3D hydrophobic interactions). The 2D interactions are depicted with different colors: pi-pi (green line), hydrogen bond (violet line) for 3D interaction hydrogen bond (purple line), pi-pi (doted sky line).

**Figure 10:**
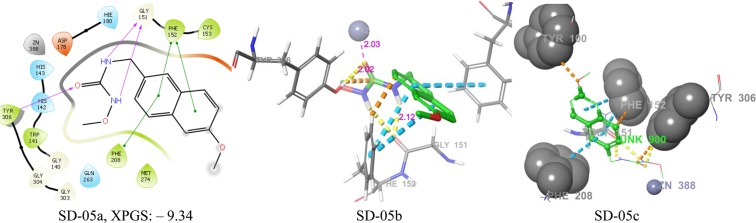
Docking poses of compound SD-05 ((**a**) 2D ligand interaction diagram like hydrogen bond donor, hydrogen bond acceptor, π-π stacking, (**b**) 3Dligandinteractions like hydrogen bond donor, hydrogen bond acceptor, π-π stacking, (**c**) 3D hydrophobic interactions). The 2D interactions are depicted with different colors: pi-pi (green line), hydrogen bond (violet line) for 3D interaction hydrogen bond (purple line), pi-pi (doted sky line).

RMSD values of different HDAC isoforms selected for molecular docking were within the acceptable range (Table 3). Superposition of docked co-ligands on its originally bound native conformation of co-ligand indicated that the quality of reproduction of a co-ligands (i.e. crystallographic) binding pose by a computational method was very good (Fig. S3). Therefore these HDAC low RMSD isoforms were used for molecular docking studies. The molecular docking studies of known selective HDAC8 inhibitors 1–32, and hits resulted after ADME filtration was performed to measure the XP glide score and also to find out interacting active site amino acid residues of HDAC8. The 2D ligand interaction diagram of 32 docked inhibitors found in the Supplementary Table S4 and analysis of 2D ligand interaction diagram are shown in Table S5. The docking analysis of 32 known selective HDAC8 inhibitors showed that the most interacting active site functionalities were divalent Zn^+2^ ion, GLY-151, PHE-152, TYR-306, PHE-208, HIS-142 including some other less interacting residues (Fig. 5). The range of XP glide scores of 32 known inhibitors were –8.1 to –11.4. The ADME filtered hits on XP glide docking with HDAC8 and best docked 20 hits with XP glide score of >9.0 were further docked with other HDACs (1, 2, 3, 4, 6). Based on the high binding affinity towards HDAC8 and less binding affinity towards other HDACs, five hits were identified as selective HDAC8 inhibitors for *in vitro* activity. The XP glide score of five selected inhibitors for different HDACs are shown in Table 4 and the 2D ligand interaction diagram of selected hits SD-01, SD-02, SD-03, SD-04, and SD-05 with different HDACs are shown in Fig. S4a– S4f. The XP glide score >9.0 of top five selected hits (Figs. 6–10) also indicated that the hits are selective towards HDAC8. The XP glide score of top five hits with other HDACs is poor compare to HDAC8 and are listed in Table 4. The most interacting active site amino acid residues of five selective HDAC8 inhibitors were Zn^+2^, GLY-151, PHE-152, HIE-180, TYR-306 including some other less interacting amino acid residues (Fig. 5). These interactions were very similar to 32 known inhibitors.

**Table 3:** The RMSD values of XP glide predicted binding modes vs co-crystal structures of respective HDACs.

HDACs	PDB ID	Co-ligand	RMSD
HDAC2	3MAX	LLX	0.2067
	5IWG	IWX	0.2204
	4LXZ	SHH	1.5526
HDAC3	4A69	I0P	1.4762
HDAC4	2VQM	HA3	1.5703
	2VQO	TFG	1.6445
	2VQJ	TFG	1.8655
HDAC6	5WPB	B8P	0.2874
	5WGM	AH7	0.8484
	5WGI	TSN	1.2314
	5W5K	K70	1.1551
HDAC8	1T64	TSN	0.3571
	5FCW	5YA	0.4290
	1T69	SHH	1.9849

**Table 4:** The XP Glide score of top five virtual hits SD-01, SD-02, SD-03, SD-04 and SD-05 against HDAC8 and glide score of these hits against other HDAC isoforms.

Virtual hits	HDAC isoforms used for docking with their PDB IDs						
	HDAC1 (4BKX)	HDAC2 (3MAX)	HDAC3 (4A69)	HDAC4 (2VQM)	HDAC6 (5WPB)	HDAC6 (5WGI)	HDAC8 (1T64)
SD-01	−6.8	−7.6	−5.5	−7.3	−4.5	−8.0	−10.2
SD-02	−7.7	−8.7	−8.2	−7.2	−5.6	−9.2	−9.3
SD-03	−7.6	−9.5	−5.6	7.3	−5.2	−8.5	−9.0
SD-04	−4.2	−6.3	−4.2	−6.7	−5.3	−7.6	−9.0
SD-05	−8.2	−8.4	−8.4	−6.8	−5.5	−8.0	−9.3

**Figure 11:**
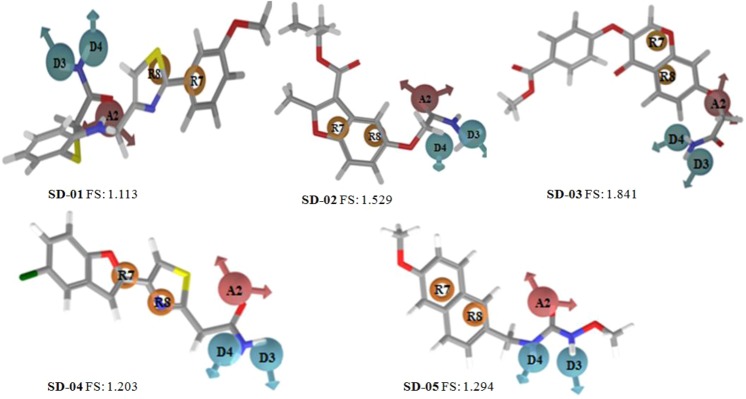
Pharmacophore matched structure of five selected HDAC8 inhibitors with fitness score (A2: hydrogen-bond acceptor, D3, D4: hydrogen bond donor and R7, R8: aromatic ring, (FS: Fitness Score).

The fitness score of SD-01, SD-02, SD-03, SD-04, and SD-05 were 1.113, 1.529, 1.203, 1.294, and 1.84,1 respectively (Fig. 11). The fitness score and pharmacophore matched structures of hits indicated that all the five hits matched five pharmacophores (ADDRR.4). The pharmacophores of SD-01 were A2 which matched with amide oxygen and it binds with Zn^+2^ (2.01 Å) and TYR-306 (2.26 Å) in the active site. The other two pharmacophores D3 and D4 matched with two hydrogens of the amide group and one of them binds with GLY-151 (1.75 Å). The other two pharmacophores R7 and R8 matched with thiazole ring and 3-methoxy phenyl ring respectively of inhibitor. The pharmacophore R8 had π-π interactions with the aromatic ring of PHE-152. Other than these common pharmacophore interactions, two π-π interactions of 2-aminothiophenol ring of the inhibitor with the aromatic ring of PHE-208 and PHE-152 were observed (Fig. 6. SD-01a,01b). The hits enclosed it with hydrophobic residues PHE-208, PHE-152, LEU-308, TRP-141, TYR-306, PRO-35 and interactions with almost three rings of hits is shown in Fig. 6. SD-01c.

The pharmacophore A2 of SD-02 matched with acetoxy hydrazide carbonyl oxygen, which binds with Zn^+2^ (2.05 Å) of the active site. The other two pharmacophores were D3 and D4 matched with hydrogens of hydrazine -NH_2_ group and one of them binds with HIS-142 (2.27 Å) and -NH- hydrogen binds with GLY-151 (2.30 Å). The pharmacophores R7 and R8 were matched with the two rings of benzofuran. The pharmacophore R8 had two π-π interactions with HIE-180 and PHE-208 aromatic rings. The inhibitor also enclosed with hydrophobic PHE-208, PHE-252, TYR-306, TYR-100. All the interactions of SD-02 are shown in Fig. 7. SD-2a, SD-2b, and SD-2c.

In SD-03, the common pharmacophore D3 and D4 matched with amide hydrogens and A2 matched with amide oxygen. The pharmacophore A2 binds with Zn^+2^ (2.13 Å) and D3, or D4 binds with GLY-151. The flavone ring matched with R7, R8, and R8 had π-π interactions with HIE-180 aromatic ring. Other than these pharmacophores, there were a hydrogen bonding interactions of benzoate ester oxygen of hits with LYS-202 and benzoate ring had π-π interactions with PHE-207. The inhibitor enclosed with hydrophobic amino acid residues PHE-152, PHE-207, PHE-208, and TYR-306 and had interactions with the R8. All the interactions of SD-03 are shown in Fig. 8. SD-03a, SD-03b, and SD-03c.

In SD-04 the hydrogen bond acceptor pharmacophore A2 matched with amide oxygen and hydrogen bond donor pharmacophore D3 and D4 matched with amide hydrogens. Pharmacophore A2 of the inhibitor binds with Zn^+2^ (2.20 Å) and TYR-306 (2.01 Å). The pharmacophore D3 or D4 binds with GLY-151 (1.98 Å). Pharmacophores R7 and R8 matched with furan ring and thiazole ring respectively, and R7 had a π-π interaction with the HIE-180 ring. Other than these pharmacophore interactions there exists one hydrogen bond acceptor interaction of furan oxygen with HIE-180. All the interactions of SD-04 are shown in Fig. 9. SD-04a, SD-04b, and SD-04c.

In SD-05, pharmacophore A2 matched with an oxygen atom of urea part of inhibitor and binds with TYR-306 (2.02 Å) and Zn^+2^ (2.03 Å). The two hydrogen bond donor pharmacophores D3 and D4 matched with hydrogens of -NH- group of substituted urea and both binds with GLY-151. The two pharmacophore R7 and R8 matched with two aromatic rings of the naphthyl group. The ring R7 had π-π interaction with PHE-152 and R8 had two π-π interactions with PHE-152 and PHE-208. The hits had a hydrophobic enclosure with TYR-100, PHE-208, PHE-152, and TYR-306. The interactions of SD-05 are shown in Fig. 10. SD-05a, SD-05b, and SD-05c. The 3D interactions of five inhibitors showed that the common pharmacophores A2, D3, D4, R7 and R8 had good interactions with active site amino acid residues in most cases.

**Figure 12:**
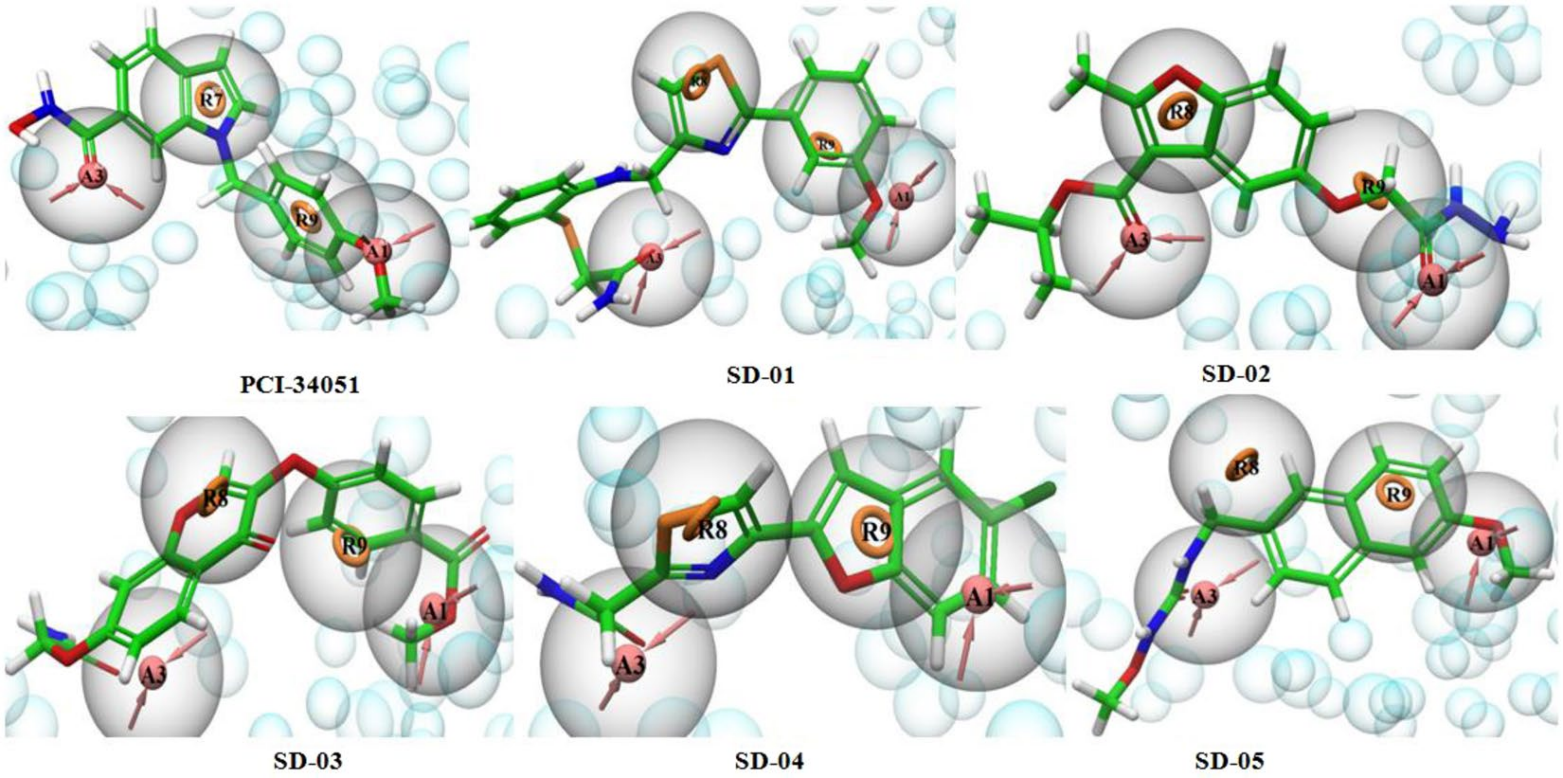
E-Pharmacophores generated from PCI-30051–1T64 docked complex and matched structures of selective five HDAC8 inhibitors. The pharmacophores are A1, A3 (hydrogen bond acceptors) and R8, R9 (ring aromatic).

The generated E-pharmacophore of best selective known HDAC8 inhibitor PCI-34051 was used for matching of five selected hits by selecting all four features matching option. In SD-01, A3 superimposed on amide oxygen, A1 matched with 3-methoxy phenyl oxygen group, R8 and R9 matched with thiazole ring and 3-methoxyphenyl ring, respectively. All the four pharmacophores of selected PCI-34051 matched with SD-01 (Fig. 12). In SD-03, pharmacophore A3 superimposed on amide oxygen and A1 superimposed on benzoate oxygen (Fig. 12). The pyran-4-one of flavone matched with R8 and benzoate ester ring matched with R9. The SD-03 also matched all the four E-pharmacophores of PCI-34051. The other three compounds did not matched all the four E-pharmacophore of PCI-34051. Therefore the matching options were reduced to three features for other hits, which resulted in SD-02, SD-04 and SD-05 matching with three pharmacophores. The pharmacophore A1 of SD-02 matched with acetoxy hydrazide carbonyl oxygen and R8 superimposed on furan ring of benzofuran. The pharmacophore A3 matched with the carbonyl oxygen of ester group and R9 had no matching (Fig. 12). The pharmacophore A1 of SD-04 and R8 of SD-05 did not match but other pharmacophores were well matched (Fig. 12).

These results showed that SD-01 and SD-03 matched all the pharmacophoric features of the E-pharmacophores and therefore may be more selective than the other three. Based pharmacophore matching, XP glide score, interacting amino acid residues, and ADME properties, virtual screening hits SD-01, SD-02, SD-03, SD-04, and SD-05 were selected for *in vitro* activity study against HDAC6 and HDAC8.

The structures of all the five identified inhibitors are new and non-hydroxamic acid as confirmed by PubChem structure search, as the activity of these compounds has not been predicted earlier for HDAC8 inhibitory activity.

### *In vitro* HDAC inhibition activity

The *in vitro* HDAC inhibitory activity results clearly showed that compound SD-01, SD-02, and SD-05 have potential HDAC8 inhibitory activities (Table 5). SD-01 is more selective towards HDAC8 over HDAC6 and SD-02 has marginal selectivity towards HDAC6 over HDAC8. The results demonstrated thatthe experimental results were similar to in silico results. The purity of the best two compounds, SD-01 and SD-02 was 94.543 and 97.630%, respectively.

**Table 5:** The observed inhibition activities of selected hits SD-01, SD-02, SD-03, SD-04 and SD-05 against HDAC-6 and HDAC8.

Sample	HDAC-6 (IC_50_ in nM)	HDAC-8 (IC_50_ in nM)
SD-01	>100	9.0
SD-02	0.62	2.7
SD-03	>100	>100
SD-04	>100	>100
SD-05	30.86	41.6

The fitness score of all the five selected hits were >1.0 and almost all the five pharmacophoric features A2, D3, D4, R7 and R8 matches the selected hits are shown in Fig. 11. Among the selected hits XP glide score of SD-01 was highest –10.2 for HDAC8 and its experimental IC_50_ value was 9.0 nm. The XP glide score of SD-01 was –4.5 and –8.0 for two pockets zinc finger domain and catalytic domain 2 respectively, of HDAC6 and its IC_50_ value was >100 nm. The XP glide score of SD-02 was –9.3 for HDAC8 and –5.6 and –9.2 respectively, for zinc finger domain (5WPB) and catalytic domain 2 (5WGI) of two HDAC6 pockets. The experimental activity of SD-02 was 2.7 nM and 0.62 nM respectively, for HDAC8 and HDAC6. The glide score for SD-05 was –8.0 and –9.3 respectively, for HDAC6 catalytic domain 2 and HDAC8. The experimental activities were 30.86 nM and 41.6 nM respectively, for HDAC6 and HDAC8. The hit SD-02 has more affinity towards the catalytic domain 2 rather than zinc finger domain. There was a good symmetry between XP glide scores and experimental activities of the best three hits SD-01, SD-02 and SD-05. The E-pharmacophores A1, A3 (hydrogen bond acceptors), R8 and R9 (ring aromatic) generated form HDAC8 and selective HDAC8 inhibitor (PCI-34051) complex, matched nicely with SD-01 (Fig. 12). Therefore, SD-01 is considerably more selective towards HDAC8 compared to HDAC6. The superimposed docked poses of all selected hits on crystallographic bound co-ligand TSN of HDAC8 (PDB ID: 1T64) in the active site showed that except SD-04, the other hits bind in a similar binding pose with TSN (Fig. S5) and experimental activity of SD-04 was low.

### Experimental

The selected virtual screening hits were obtained from Enamine and their structures were further confirmed by mass spectra (Fig. S8a–S8e) and purity of the compound was confirmed by analytical HPLC (Fig. S7a–S7e). The mass spectra of SD-01: HRMS (ES+), exact calc. mass for C_19_H_20_N_3_O_2_S_2_ [M + H]^+^, 386.0997. Found m/z 386.0988. The mass spectra of SD-02: HRMS (ES+), exact calc. mass for C_15_H_19_N_2_O_5_ [M + H]^+^, 307.1294. Found m/z 307.1288. The mass spectra of SD-03: HRMS (ES+), exact calc. mass for C_19_H_16_NO_7_ [M + H]^+^, 370.0927. Found m/z 370.0919. The mass spectra of SD-04: HRMS (ES+), exact calc. mass for C_13_H_10_N_2_O_2_SCl [M + H]^+^, 293.0152. Found m/z 293.0146. The mass spectra of SD-05: HRMS (ES+), exact calc. mass for C_14_H_16_N_2_O_3_Na [M + Na]^+^, 283.1059. Found m/z 283.1051.

The purity of compounds SD-01, SD-02, SD-03, SD-04, and SD-05 were 94.543, 97.630, 93.014, 83.471 and 80.034%, respectively. The HPLC chromatogram is available in supporting information Fig. S7a– S7e.

### HDAC6 and HDAC8 activity assay

HDAC 6 and 8 activities were measured according to the manufacturer’s protocol from Sigma (Cat# CS1010). Briefly, to 5 ng of HDAC6 or 8 enzymes, compounds (1, 10, 50 and 100 nM) were added and incubated in the presence of substrate (Boc-Lys(Ac)-AMC) for 30 min at 30 °C. One μL developer was added to this mix and further incubated for 15 min and the fluorescent group liberated from the cleaved substrate, which is proportional to the deacetylase activity was measured using a fluorescence plate reader at Ex-350 nm; Em-440 nm. Trichostatin A was used as a reference inhibitor. The Perkin Elmer multimode reader, model-Enspire 2300 was used for fluorescence plate reader.

On the basis of in silico ligand, structure-and ADME filtration tools, a series of five HDAC8-selective non-hydroxamate hits SD-01, SD-02, SD-03, SD-04, and SD-05 were identified from the commercial database. *In vitro* HDAC inhibitory studies of identified leads demonstrated that SD-01 has potential inhibitory activity and good selectivity for HDAC8 over HDAC6. SD-02 has potential activity but marginal selectivity for HDAC6. The results of the present studies encouraged us for further studies on SD-01 to develop a potential candidate for isoform selective HDAC8 drug for the treatment of HDAC8-overexpressed cancers. The interesting facts are all the identified hits are structurally diverse and are from non-hydroxamic acid series. The common pharmacophore information: one hydrogen bond acceptor, two hydrogen bond donor and two ring aromatic and their interactions with active site amino acid residues and molecular docking results of 32 known selective HDAC8 inhibitors which were used in the present study may be useful for further design of isoform-selective HDAC8 inhibitors. The ADME properties revealed that the identified hits can be further developed as good oral drug candidates. These *in-vitro* identified leads may be used for designing anti-cancer chemo-therapeutics related to over expression of HDAC8 and HDAC6. Further *in vitro* study on other HDACs will be carried out in a future. This method may be useful for identification of isoform-selective HDACs inhibitors and the E-pharmacophore matched hits may be more selective towards HDACs isoform.

## Materials and Methods

### Dataset

**Figure 13:**
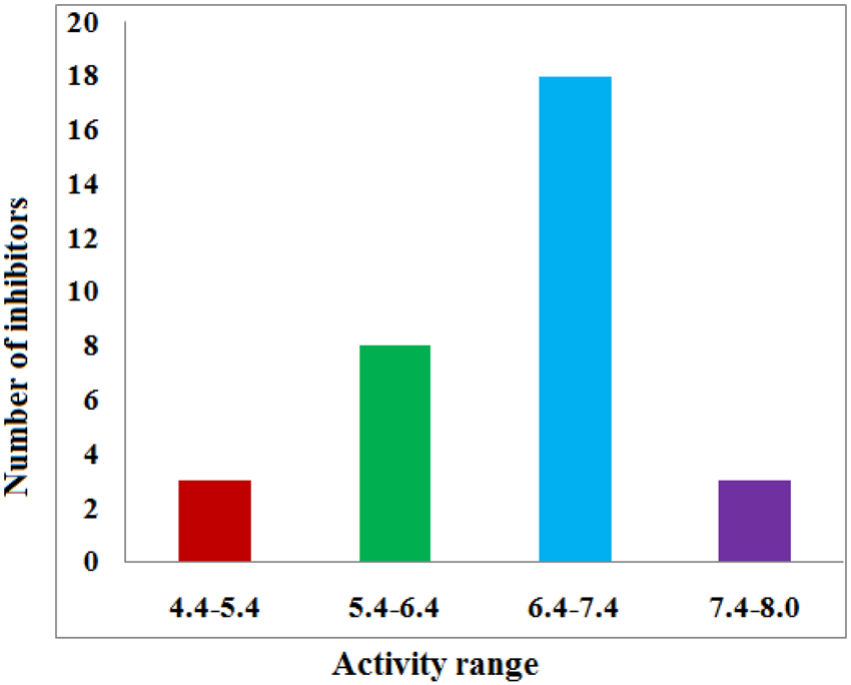
Distribution of 32 known selective HDAC8 inhibitors with a different activity range.

Thirty two selective HDAC8 inhibitors have been chosen from the existing literature^4,33–35^ with inhibitory activity data (IC_50_ in μM) (Fig. 1). The IC_50_ values of the compounds were converted to their pIC_50_ values consisting of some high active, medium active and low active molecules. The distribution of activity data of known inhibitors and the number of compounds is shown in Fig. 13 which confirms the data span over 4 order magnitudes (4.469–8.000). The Phase database, a database of commercially available compounds contains 4.3 × 10^6^ compounds (only first conformer) with a unique identifier, CACPD2011aCode was chosen for virtual screening^36^. The crystal structure of HDAC1 (PDB ID: 4BKX: resolution: 3.0 Å)^37^, HDAC2 (3MAX, 2.05 Å)^38^, HDAC3 (4A69, 2.06 Å)^39^, HDAC4 (2VQM, 1.8 Å)^40^, HDAC6 (5WPB, 1.55 Å)^41^, HDAC8 (1T64, 1.90 Å)^42^ was retrieved from RCSB Protein Data Bank (http://www.rcsb.org/pdb/) which was used for molecular docking study. All the computational calculations were performed on HP Z820 Workstation with CentOS 6.3.

### Identified hits

The hits SD-01: C_19_H_19_N_3_O_2_S_2_, [2-((2-(((2-(3-methoxyphenyl)thiazol-4-yl)methyl)amino)phenyl)thio)acetamide, Phase Databank ID: CACPD2011a-0001983707, Enamine ID: T6550042], SD-02: C_15_H_18_N_2_O_5_ [isopropyl 5-(2-hydrazineyl-2-oxoethoxy)-2-methylbenzofuran-3-carboxylate, Phase Databank ID: CACPD2011a-0001289047, Enamine ID: Z374510610], SD-03: C_19_H_15_NO_7_ [methyl 4-((7-(2-amino-2-oxoethoxy)-4-oxo-4H-chromen-3-yl)oxy)benzoate, Phase Databank ID: CACPD2011a-0001268860, Enamine ID: Z374511496], SD-04: C_13_H_9_ClN_2_O_2_S [2-(4-(5-chlorobenzofuran-2-yl)thiazol-2-yl)acetamide, Phase Databank ID: CACPD2011a-0001271129, Enamine ID:T6218428], SD-05:C_14_H_16_N_2_O_3_ [1-methoxy-3-((6-methoxynaphthalen-2-yl)methyl)urea, Phase Databank ID: CACPD2011a-0000738295, Enamine ID: T5848596] were purchased from ENAMINE Ltd., 78 Chervonotkatska Street, 02660 Kyiv, Ukraine. The ^1^H-NMR spectra of best three hits SD-01, SD-02 and SD-03 are attached (Fig. S6a, S6b and S6c).

### Ligand preparation

All the structures of the inhibitors were drawn using 2D sketcher of Maestro 9.6 and then converted to their corresponding 3D structures. These inhibitors were geometrically refined using Ligprep module of Schrodinger^43^. During ligand preparation, the OPLS_2003 force field was used for energy minimization. Ligprep generates single, low energy, 3D structures, retaining its original state of chiralities and ionization for each input structure.

### Protein preparation

The X-ray crystal structures of different PDB IDs of HDACs namely 4BKX, 3MAX, 4A69, 2VQM, 5WPB, 1T64 were prepared using ‘Protein Preparation Wizard’ workflow in Maestro 11.4 (Schrodinger Inc.). During protein preparation, water molecules were removed and then hydrogens were added to the protein and co-ligand. The energy of the complexes was then minimized until the RMSD between the minimized structure and the starting structure reached 0.30 Å, using the OPLS_2003 force field. The receptor grid box of 15 Å cube was generated by selecting the co-ligand of the active site except for HDAC1. The HDAC1 grid box was generated selecting active site amino acids ASP-264, ASP-176, and HIS-178^35^. The receptor grid of HDAC8 (PDB ID: 1T64) used in the present study was prepared earlier^32^.

### Generation of common pharmacophore hypothesis

Phase module of Schrodinger^44^ provides six inbuilt pharmacophore features: hydrogen bond acceptor (A), hydrogen bond donor (D), hydrophobic group (H), negatively charged group (N), positively charged group (P), and aromatic ring (R). The common pharmacophores were identified using a tree-based partitioning technique which groups together similar pharmacophores according to their inter-site distances. Five featured pharmacophore hypotheses were selected and subjected to stringent scoring function analysis. The set of the generated hypotheses with their scoring values are summarized in Table S6. The distance and angle of pharmacophore features for all hypotheses are listed in the supplementary material Table S7 and Table S8. The best hypotheses ADDRR.4 consisting of five features are: one hydrogen bond acceptors (A2), two hydrogen bond donors (D3, D4), and two aromatic rings (R7 and R8) are shown in Fig. 2.

### The building of 3D QSAR models

The PHASE provides the option of building a 3D QSAR model with the selected pharmacophore hypothesis. Top ranking hypotheses based on survival score (representing a weighted combination of the vector, site, volume scores) and survival – inactive score, ADDRR.4, AADRR.4, and AADDR.12, were subjected to 3D QSAR model building. During the model building randomly selected 70% molecules were kept in training set and atom based model was generated by keeping 1 Å grid spacing. Thus the maximum of PLS factors which can be used is N/5, where N symbolizes the number of ligands present in the training set. The model with a PLS factor four were considered as the best statistical models. The 3D QSAR models generated from hypotheses ADDRR.4 has admirable statistical parameters (Table 1) and was used for virtual screening of the database.

### Validation of 3D QSAR model

Validation of 3D QSAR model is a crucial part, especially when the model is used for virtual screening or prediction of activity for external data set^45^. The performance of a model was measured by its predictive ability. The experimental activity of external selective HDAC8 inhibitors (**1′**–**22′)** retrieved from literature^4,6,17,34,46–48^ with their structures and predicted their activity using built 3D QSAR model (Fig. 4). These molecules were not included in the model building but used for model validation. The chemical features of the ligand structures are mapped to a cubic 3D grid by the 3D QSAR models.

### Virtual screening of the database

**Figure 14:**
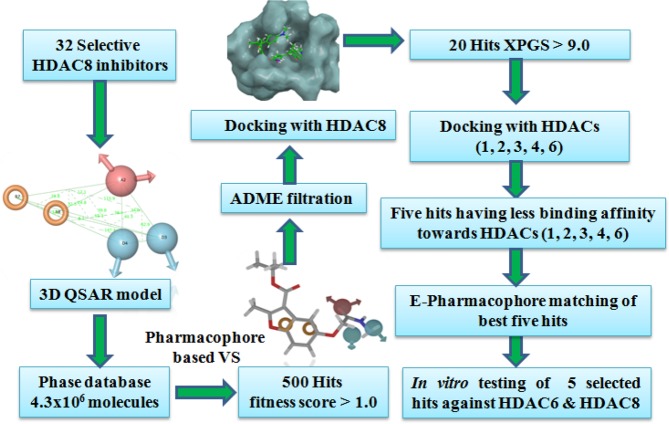
Virtual screening workflow for identification of novel selective HDAC8 inhibitors.

Pharmacophore-based virtual screening (VS) of the database has been used to retrieve selective HDAC8 inhibitors. The 3D QSAR model built from pharmacophore ADDRR.4 was used to search the Phase database. The virtual screening workflow for the identification of HDAC8 inhibitors is presented in Fig. 14. The compounds of the database matching with minimum of four pharmacophoric features were identified through the ligand pharmacophore mapping process. The number of 500 top scored hits with fitness score ≥ 1.0 resulted from this step were subjected for ADME filtration.

### ADME study

The QikProp 3.5^49^, a prediction program designed by Prof. William L. Jorgensen was used to calculate ADME properties. It is a quick, accurate and predicts physically significant descriptors and pharmaceutically relevant properties of organic compounds. It has been widely used as a filter for molecules that would be expected to be further developed in drug design programs. QikProp provides ranges of values for comparing particular molecular properties of organic molecules with those of 95% of recognized drugs. The number of 500 hits resulted from virtual screening were filtered to find out a drug like hits and ADME properties of five finally selected hits with their acceptable range are listed in Table 2.

### Validation of docking

The validation of the docking was measured by calculating the root mean square deviation (RMSD). Superimposing the original crystallographic bound conformation of co-ligand and its predicted docked conformations were expressed as RMSD. Lower the value of RMSD, higher the accuracy of docking and RMSD values less than 1.5 Å or 2.0 Å depending on ligand size are considered to have performed successfully^50^. Amongst the several HDACs, the lowest RMSD PDB ID of each HDACs was selected for molecular docking studies.

### Molecular docking studies

The hits from ADME filtration and 32 known selective HDAC8 inhibitors were subjected for molecular docking study with HDAC8 to predict the binding affinity towards HDAC8 using Glide module of Schrodinger^51–54^. The best 20 hits with XP glide score >9.0 were further subjected to molecular docking studies with HDAC1, HDAC2, HDAC3, HDAC4, and HDAC6 to identify isoform selectivity of these hits.

### E-Pharmacophoregeneration

The best selective known HDAC8 inhibitor PCI-34051(**1**) [N-hydroxy-1-(4-methoxybenzyl)-1H-indole-6-carboxamide] docked with a previously prepared grid of 1T64 proteins and energy-optimized structure-based pharmacophore (E-pharmacophore) were generated by selecting protein-PCI-34051 docked complex as per protocol^55,56^. The generated pharmacophore of PCI-34051 could effectively map all the important pharmacophoric features of PCI-34051- A1, A3, R7, and R9. The pharmacophore A1 and R9 superimposed on the oxygen atom of the methoxy group and benzene ring of methoxybenzyl group respectively whereas R7 and A3 superimposed on indole five member ring and hydroxamic acid carbonyl oxygen respectively, of PCI-34051. These generated pharmacophores were used for matching the finally selected five HDAC8 inhibitors.

## Supporting Information

Supporting Information Final
