# Supplementary material for: Discovery of novel potential selective HDAC8 inhibitors by combine ligand-based, structure-based virtual screening and in-vitro biological evaluation
Source: Sci Rep. 2019 Nov 20;9:17174. doi: 10.1038/s41598-019-53376-y (PMC6868012; doi:10.1038/s41598-019-53376-y)
Supplement: Supplementary file 1 — Supporting Information Final [file 41598_2019_53376_MOESM1_ESM.pdf]

Supplementary Information

Discovery of novel potential selective HDAC8 inhibitors by combine ligand-based, structure-based virtual screening and *in-vitro* biological evaluation

Sudhan Debnath[1*], Tanusree Debnath[1], Samhita Bhaumik[2], Swapan Majumdar[3], Arunasree M.Kalle[4#], Vema Aparna[5†]

[1]Department of Chemistry, MBB College, Agartala, Tripura, 799004, India

[2]Department of Chemistry, Women's College, Agartala, Tripura, 799001, India

[3]Department of Chemistry, Tripura University, Suryamaninagar, Agartala, Tripura, 799022, India

[4#]Department of Animal Biology, School of Life Sciences, University of Hyderabad, Hyderabad TS – 500046, India

[5†]Sree Chaitanya Institute of Pharmaceutical Sciences, Karimnagar– 505 527, Andhra Pradesh, India

***Corresponding author:** Sudhan Debnath, Department of Chemistry, MBB College, Agartala, Tripura, 799004, India; Tel: 09436518210; Email: bcsdebnath@gmail.com

Arunasree M Kalle, Department of Animal Biology, School of Life Sciences, University of Hyderabad, Hyderabad TS – 500046, India

Vema Aparna, [5]Sree Chaitanya Institute of Pharmaceutical Sciences, Karimnagar– 505 527, Andhra Pradesh, India

Sl. No	**CONTENTS**	Page No
1	**Table S1**. Structure of known selective HDAC8 inhibitors with pIC_{50} values retrieved from literature used for validation of the model	3-5
2	**Table S2**. The experimental and predicted activity of the test and training set molecules	6
3	**Table S3** Predicted remaining ADMET properties of five identified selected HDAC8 hits and their recommended range	7
4	**Table S4**. 2D ligand interaction diagram of 32 known selective HDAC8 Inhibitors	8-12
5	**Table S5**. Number of interactions with different active site amino acid residues and Zn^{+2} metal	13
6	**Table S6**. Pharmacophore hypothesis with their scoring values	14
7	**Table S7**. The distance between the pharmacophoric features	14
8	**Table S8**. The angle between the pharmacophoric features	15
9	**Figure S1.** The structural classification of 32 selective known HDAC8 inhibitors A–F , used for model generation	15-17
10	**Figure S2**. Classification of HDAC isoforms	17
11	**Figure S3**. Superposition of the docked co-ligands (ash coloured) on their respective crystallographic bound conformation (green) of different HDACs (HDAC2, HDAC3, HDAC4, HDAC6, HDAC8).	18-19
12	**Figure S4a**. 2D-ligand interaction diagram of selected inhibitors SD-01, SD-02, SD-03, SD-04 and SD-05 with HDAC1	20
13	**Figure S4b**. 2D-ligand interaction diagram of selected inhibitors SD-01, SD-02, SD-03, SD-04 and SD-05 with HDAC2	21
14	**Figure S4c**. 2D-ligand interaction diagram of selected inhibitors SD-01, SD-02, SD-03, SD-04 and SD-05 with HDAC3	22
15	**Figure S4d**. 2D-ligand interaction diagram of selected inhibitors SD-01, SD-02, SD-03, SD-04 and SD-05 with HDAC4	23
16	**Figure S4e**. 2D-ligand interaction diagram of selected inhibitors SD-01, SD-02, SD-03, SD-04 and SD-05 with HDAC6 (PDB ID: 5WPB)	24
17	**Figure S4f**. 2D-ligand interaction diagram of selected inhibitors SD-01, SD-02, SD-03, SD-04 and SD-05 with HDAC6 (PDB ID: 5WGI)	25
18	**Figure S5**. Visualization of binding mode of SD-01 (a), SD-02 (b), SD-03 (c), SD-04 (d), and SD-05 (e) by superposition on crystallographic bound co-ligand TSN of HDAC8 (PDB ID: 1T64) in its active site	26
19	**Figure S6a**. ^{1}H-NMR of compounds SD-01	26
20	**Figure S6b**. ^{1}H-NMR of compounds SD-02	27
21	**Figure S6c**. ^{1}H-NMR of compounds SD-05	27
22	**Figure S7a-7e**. HPLC purity of compounds SD-01, SD-02, SD-03, SD-04 and SD-05	28,30,32,34,36
23	**Figure S8a-8e**. Mass spectra of compounds SD-01, SD-02, SD-03, SD-04 and SD-05	29, 31, 33, 35,37
24	**References S**	38

**Table S1**. Structure of known selective HDAC8 inhibitors with pIC_{50} values retrieved from literature used for validation of model	IC_{50} (μM)	pIC_{50}	Pred pIC_{50}
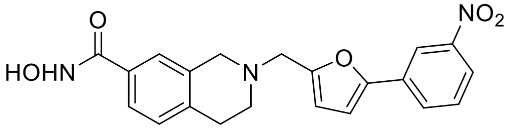 1'	0.03 [6]	7.523	6.727
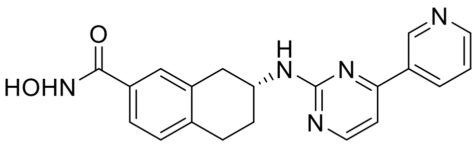 2'	0.08 [6]	7.097	6.572
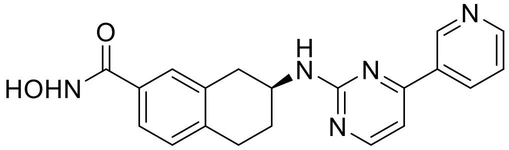 3'	0.39 [6]	6.409	6.436
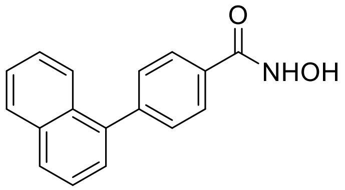 4'	0.30 [4]	6.523	6.059
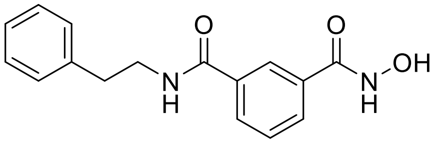 5'	0.12 [4]	6.921	6.679
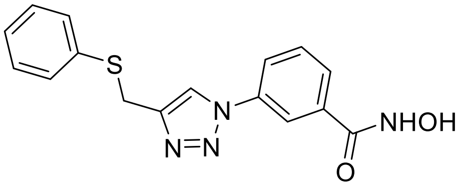 6'	0.053 [4]	7.276	6.946
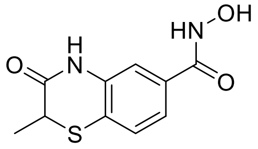 7'	0.97 [4]	6.013	6.458
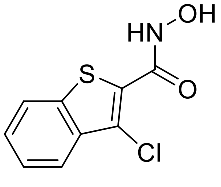 8'	3.10 [4]	5.509	6.66

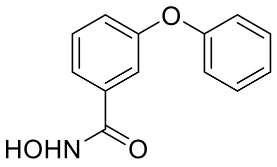 **9'**	6.60 [17]	5.18	6.255
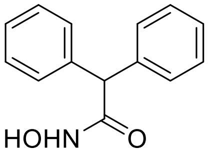 **10'**	66.0 [17]	4.18	4.63
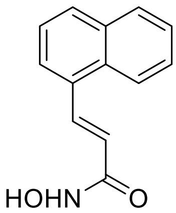 **11'**	0.70 [17]	6.155	6.955
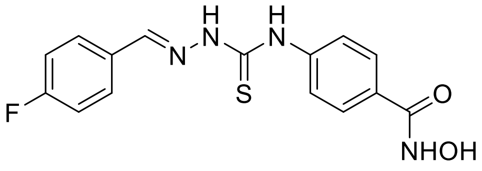 **12'**	19.7 [34]	4.706	5.841
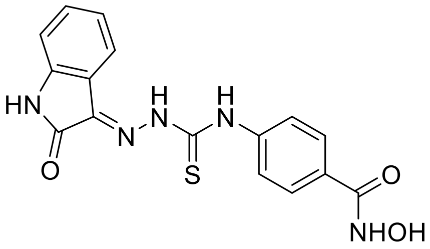 **13'**	15.7 [34]	4.804	5.842
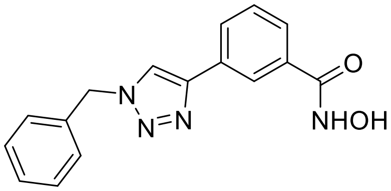 **14'**	0.35 [46]	6.456	6.175
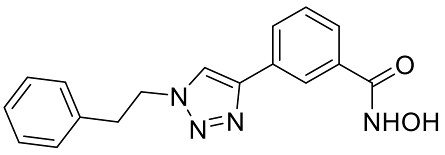 **15'**	0.18 [46]	6.745	6.769
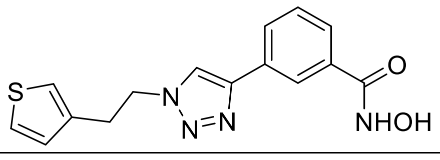 **16'**	0.10 [46]	7	6.4

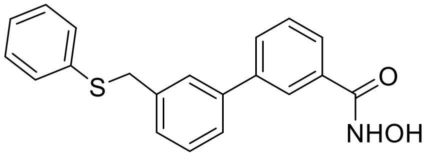 17'	1.40 [47]	5.854	5.586
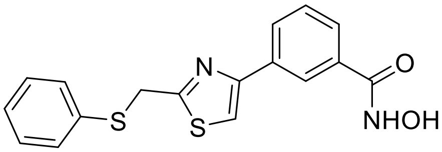 18'	0.15 [47]	6.824	5.922
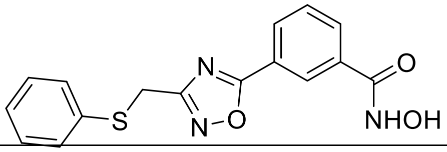 19'	0.12 [47]	6.921	6.071
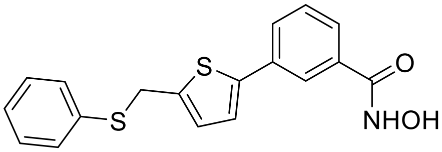 20'	0.22 [47]	6.658	5.968
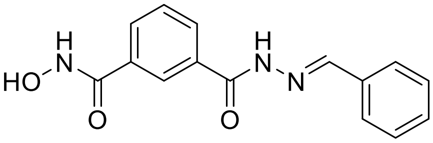 21'	0.052 [48]	7.284	6.33
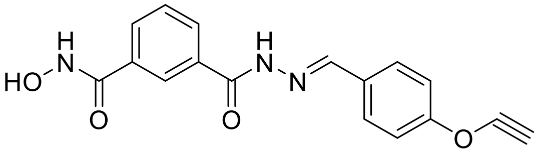 22'	0.029 [48]	7.538	6.496

**Table S2**. The experimental and predicted activity of the test and training set molecules									
Ligand	QSAR Set	Activity	Predicted Activity	Fitness	Ligand	QSAR Set	Activity	Predicted Activity	Fitness
01	training	8.000	8.02	2.49	17	test	6.463	6.30	1.89
02	training	7.620	7.57	3.00	18	training	6.456	6.48	1.83
03	training	7.420	7.44	1.80	19	training	6.452	6.45	1.88
04	training	7.398	7.42	1.56	20	training	6.398	6.40	1.14
05	test	7.398	7.16	1.66	21	test	6.385	6.48	1.99
06	training	7.310	7.28	1.72	22	training	6.337	6.32	1.87
07	training	7.301	7.28	1.68	23	training	6.102	6.13	1.84
08	training	7.000	6.97	1.99	24	test	6.097	6.00	1.69
09	test	6.854	7.03	1.49	25	training	5.971	5.98	0.91
10	training	6.824	6.85	1.62	26	test	5.553	6.04	1.53
11	test	6.721	6.21	1.20	27	training	5.410	5.42	1.55
12	training	6.721	6.72	1.84	28	training	5.155	5.15	1.35
13	training	6.678	6.70	1.80	29	training	4.469	4.46	1.68
14	training	6.638	6.66	1.89	30	training	4.633	4.63	1.91
15	test	6.523	6.53	1.88	31	training	7.009	7.02	1.13
16	training	6.469	6.44	1.89	32	test	7.155	6.71	1.78

**Table S3** Predicted remaining ADMET properties of five identified selected HDAC8 hits and their recommended ranges							
Inhibitors	amide	rotor	rtvFG	dipole	SASA	FOSA	FISA
SD-01	1	7	0	7.334	622.583	155.65	78.277
SD-02	0	6	1	4.336	582.527	270.18	182.682
SD-03	1	6	1	9.783	652.355	133.395	207.921
SD-04	1	2	0	7.466	518.84	46.843	124.908
SD-05	1	4	1	8.277	547.423	236.948	94.638
RV	0-1	0-15	0-2	1-12.5	300-1000	0-750	7-330
molecule	PISA	WPSA	volume	dip^2/V	ACxDN^.5/SA	glob	QPpolrz
SD-01	318.376	70.281	1134.281	0.047419	0.017388	0.84485	37.786
SD-02	129.666	0	1001.415	0.018777	0.018583	0.83098	31.308
SD-03	311.038	0	1115.825	0.085776	0.017885	0.79752	37.619
SD-04	232.802	114.287	855.346	0.065166	0.012266	0.83989	29.016
SD-05	215.837	0	899.522	0.07616	0.011496	0.82322	29.338
RV	0-450	0-175	500-2000	0-0.13	0-0.05	0.75-0.95	13-70
Inhibitors	QPlogPC16	QPlogPoct	QPlogPw	CIQPlogS	QPlogHERG	QPlogKp	IP(eV)
SD-01	12.737	20.979	15.413	-4.81	-4.186	-1.169	8.241
SD-02	10.351	18.128	12.472	-3.468	-4.885	-3.853	9.12
SD-03	12.683	21.201	17.771	-3.771	-4.917	-3.68	9.292
SD-04	9.81	16.145	13.469	-3.522	-3.858	-2.809	8.891
SD-05	9.063	15.523	12.17	-2.676	-4.047	-2.119	8.619
RV	4-18	8-35	4-45	-6.5-0.5	below -5	-8.0- -10	7.9-10.5
Inhibitors	EA(eV)	#metab	QPlogKhsa	SAamideO	PSA	NandO	#in56
SD-01	1.477	8	-0.18	33.335	76.439	5	17
SD-02	0.646	4	-0.15	0	118.849	7	9
SD-03	0.781	3	-0.52	43.51	138.514	8	16
SD-04	1.24	4	-0.347	41.987	74.544	4	14
SD-05	0.895	2	-0.324	35.019	71.385	5	10
RV	-0.9-1.7	1-8	-1.5-1.5	0-35	7-200	2-15	

RV: Recommended values; For all the five molecules the values are same and is #amine: 0 (0-1); #amidine: 0 (0-1); #acid: 0 (0-1); SAfluorine: 0 (0-100); #in34: 0; #noncon: 0

Table S4. 2D ligand interaction diagram of 32 known selective HDAC8 Inhibitors

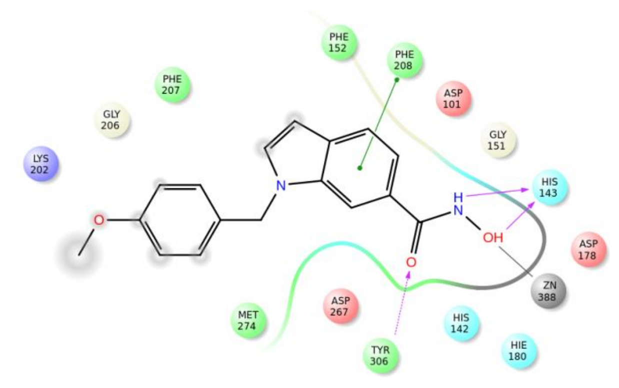

1 (-8.8)

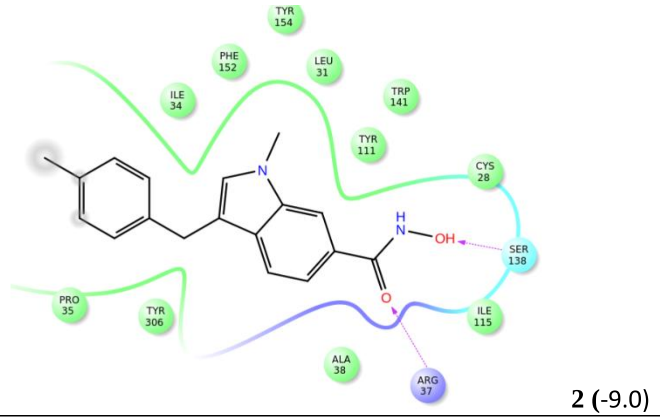

2 (-9.0)

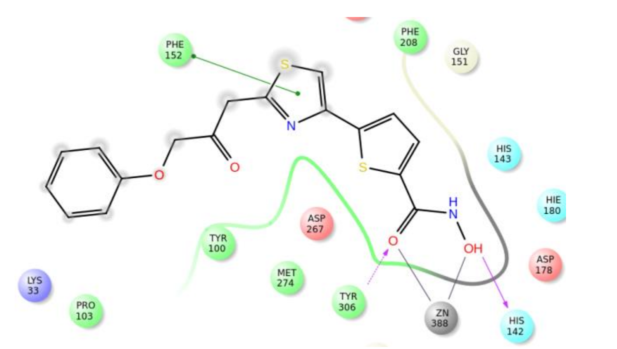

3 (-10.0)

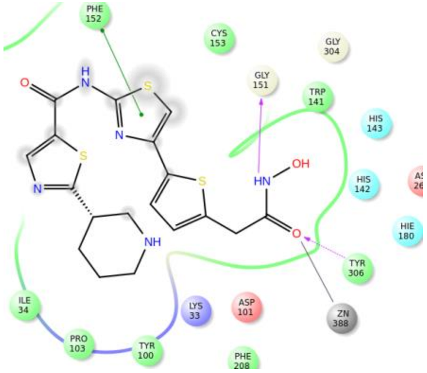

4 (-9.4)

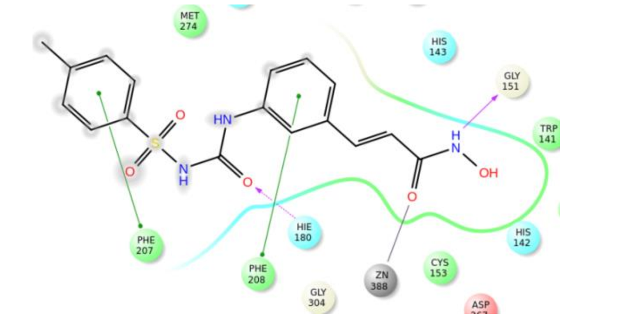

5 (-10.0)

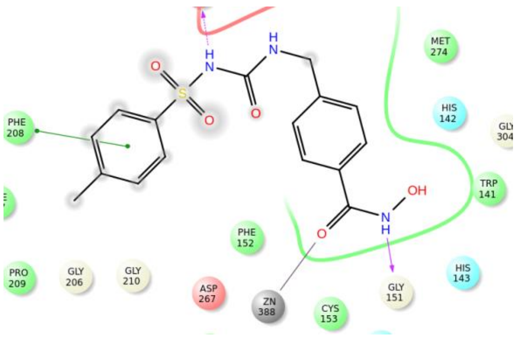

6 (-10.6)

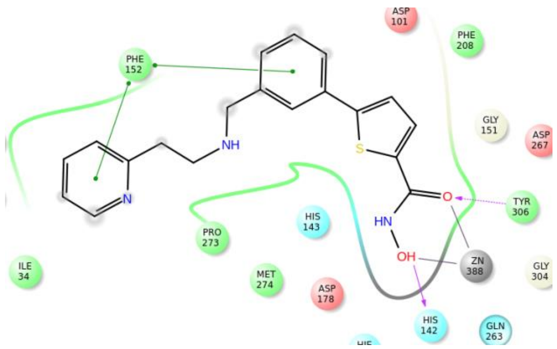

7(-10.9)

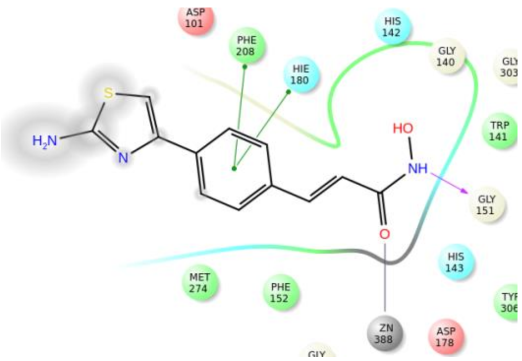

8 (-8.3)

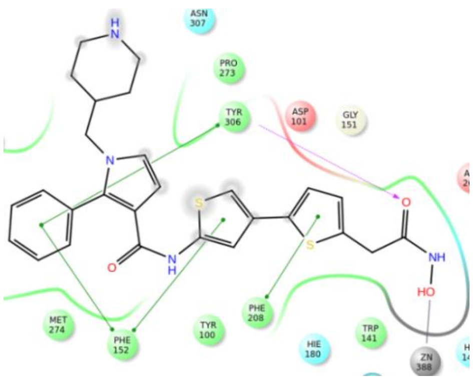

9 (-11.1)

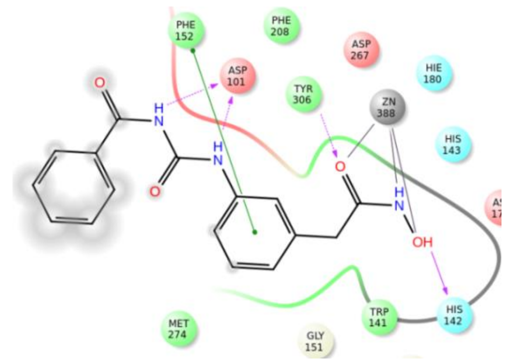

10 (-9.9)

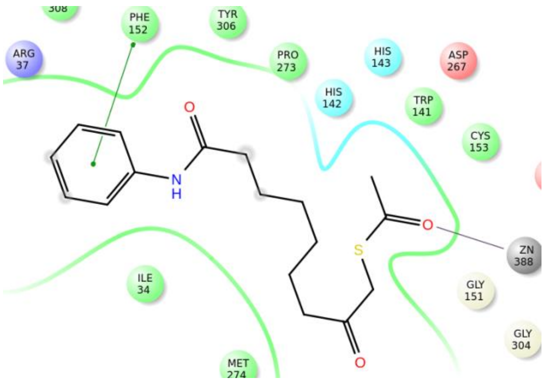

11 (-9.1)

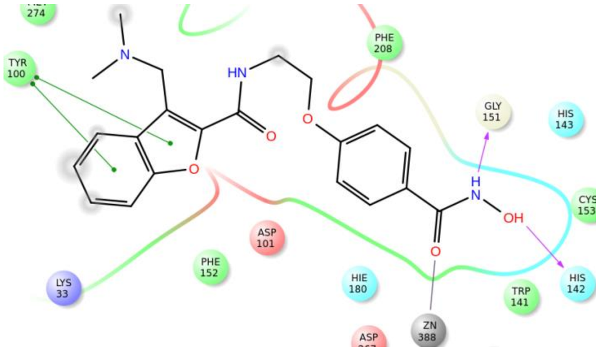

12 (-10.6)

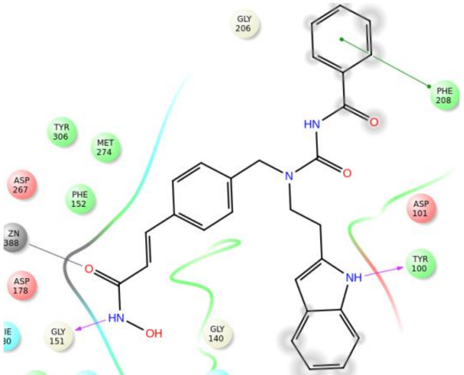

13 (-11.4)

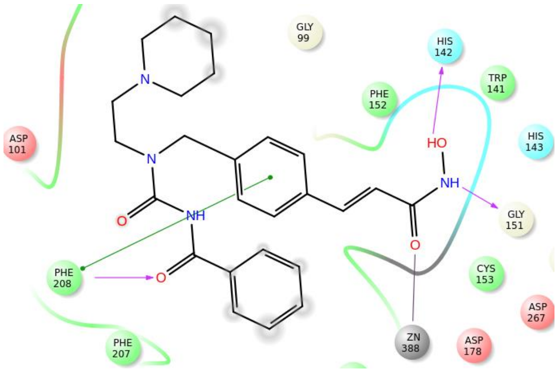

14 (-10.1)

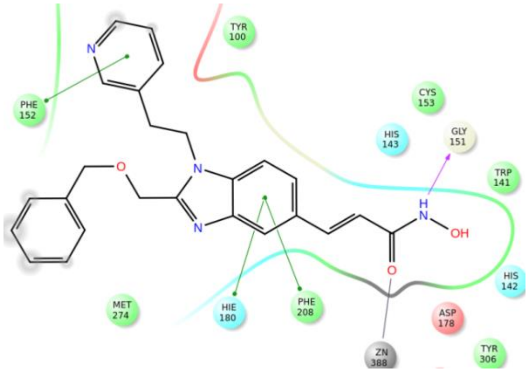

15 (-9.7)

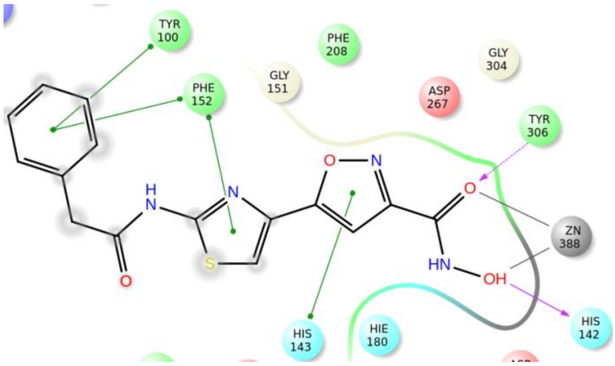

16 (-9.6)

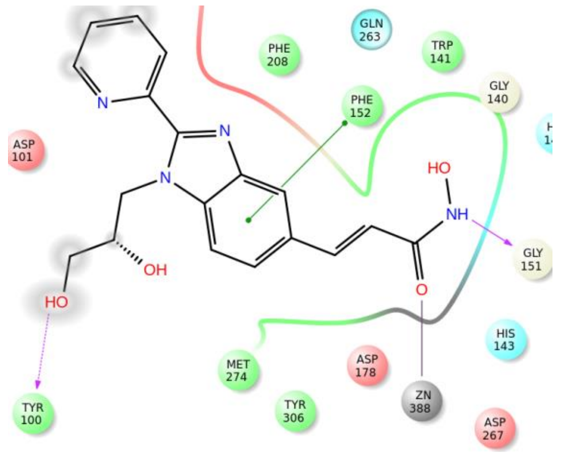

17 (-10.3)

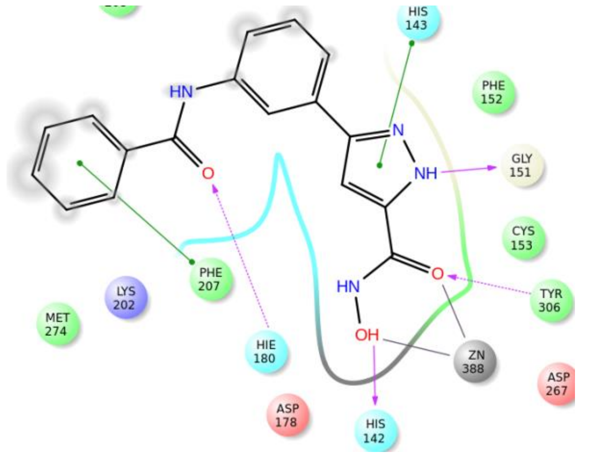

18 (-8.7)

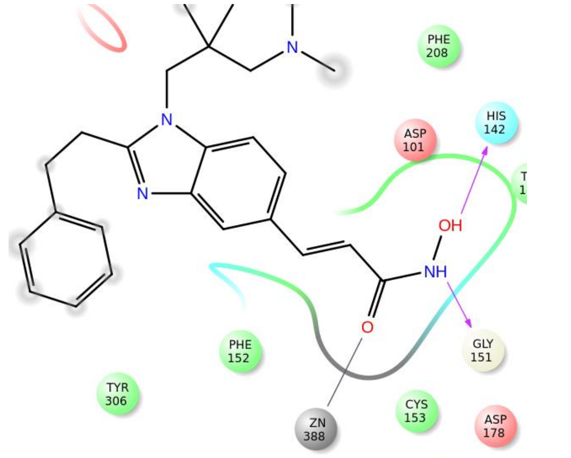

19 (-10.1)

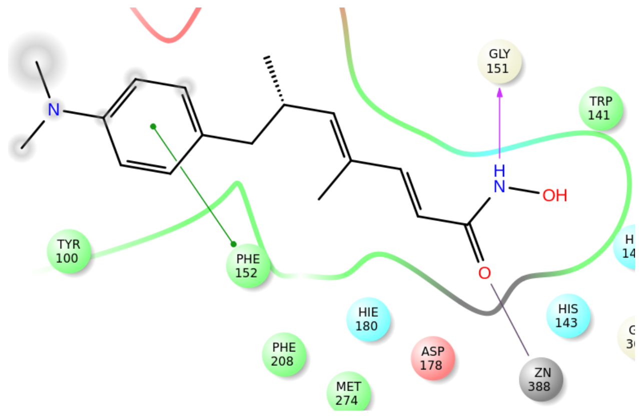

20 (-10.2)

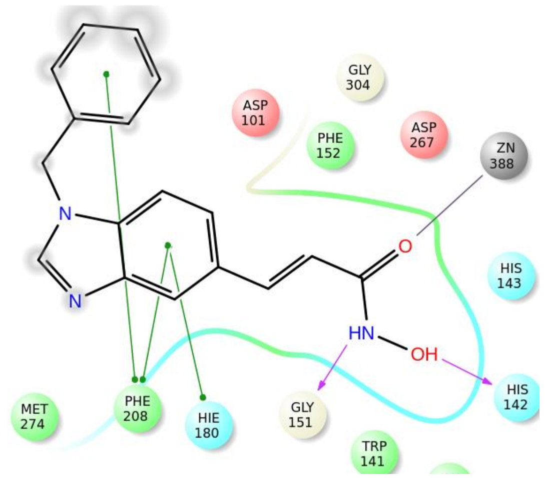

21(-9.4)

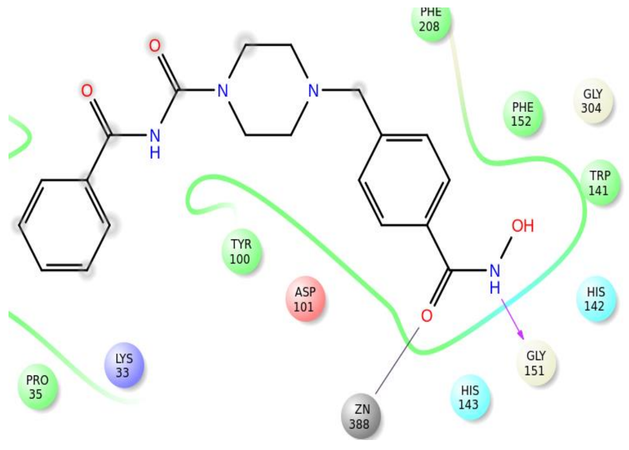

22(-11.2)

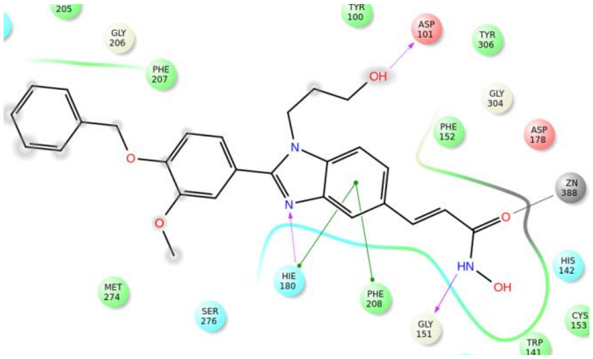

23 (-10.0)

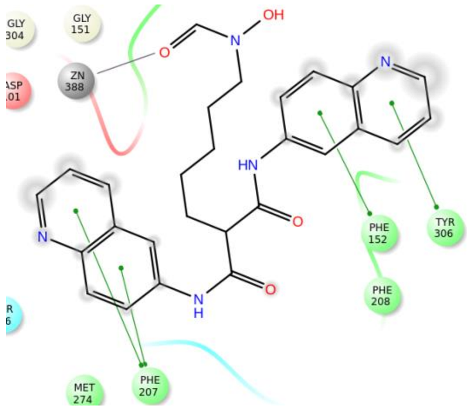

24 (-8.9)

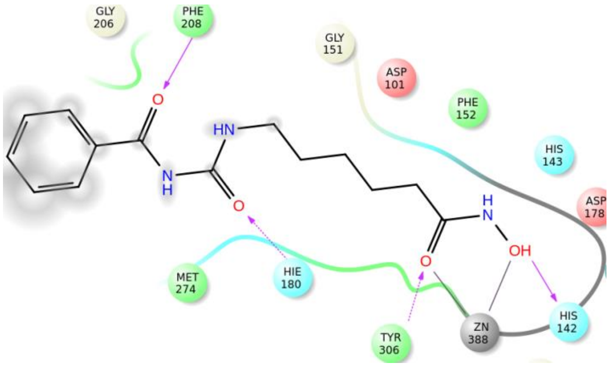

25 (-9.4)

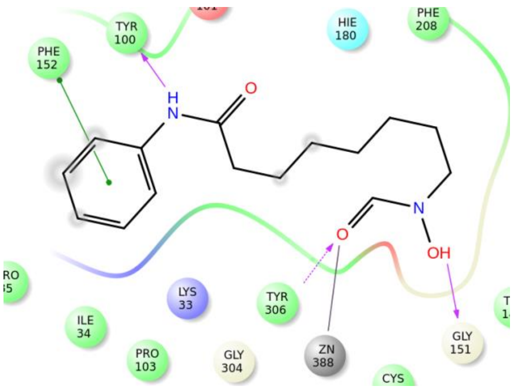

26 (-8.4)

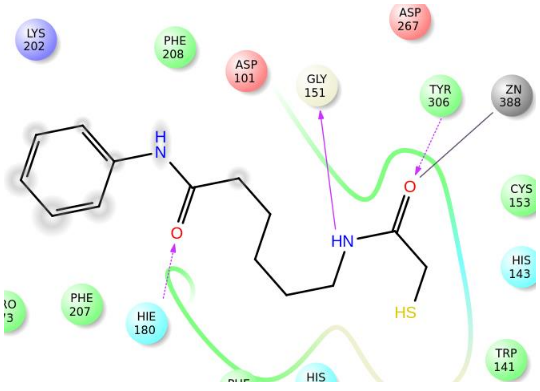

27 (-9.0)

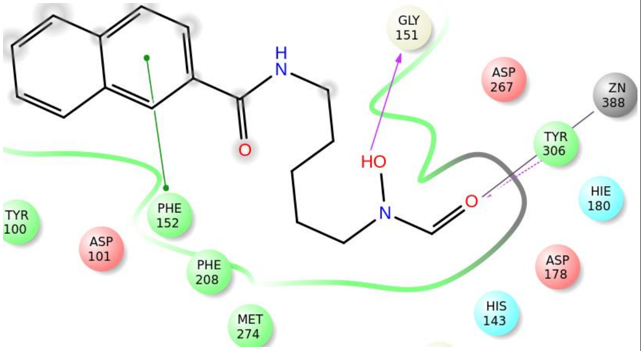

28 (-8.5)

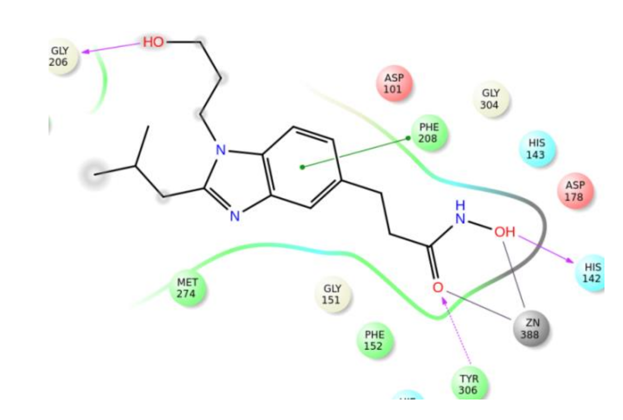

29 (-10.3)

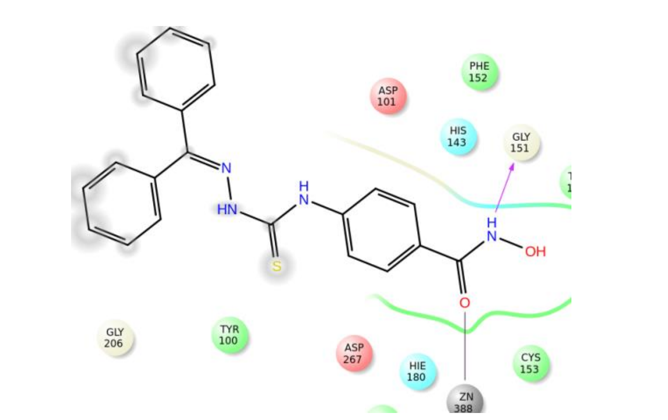

30 (-10.2)

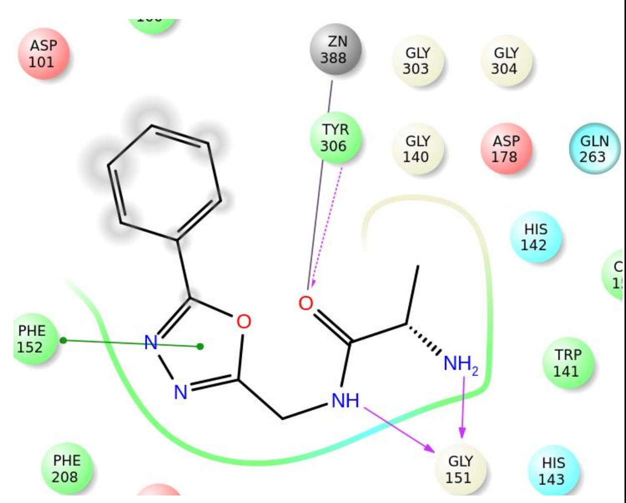

31(-8.1)

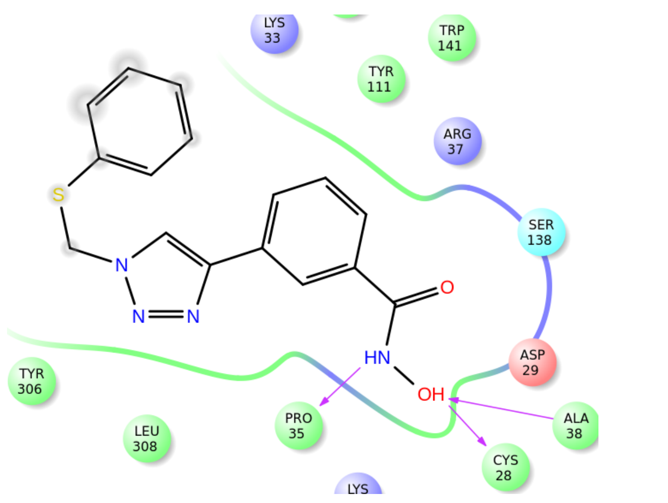

32 (-8.7)

**Table S5**. Number of interactions with different active site amino acid residues and Zn^{+2} metal										
32 known inhibitors						5 identified lead molecules				
Residues	HBD	HBA	HPB	O-H	O=C	HBD	HBA	HPB	O-H	O=C
CYS-28	1									
PRO-35	1									
ARG-37		1								
ALA-38		1								
TYR-100	2	1	3							
ASP-101	5									
SER-138		1								
HIS-142	11					1				
HIS-143	2		2							
GLY-151	21	1				5				
PHE-152			17					3		
HIE-180		5	4				2	3		
GLY-206	1									
PHE-207			4					1		
PHE-208		2	12							
TYR-306		14	2				1	1		
Zn-388				9	28					5

**Table S6**. Pharmacophore hypothesis with their scoring values										
ID	Survival	Survival -inactive	Post-hoc	Site	Vector	Volume	Selectivity	Matches	Activity	Inactive
ADDRR.4	5.139	3.298	3.493	1.00	1.00	0.495	1.546	2	7.62	1.841
AADRR.4	4.939	3.177	3.492	1.00	1.00	0.495	1.347	2	8.00	1.762
AADDR.12	4.310	2.588	2.676	0.46	0.855	0.360	1.535	2	8.00	1.722
AADDR.11	4.293	2.632	2.676	0.46	0.855	0.360	1.518	2	7.42	1.661
AADDR.15	4.214	3.186	2.539	0.42	0.774	0.345	1.574	2	8.00	1.028
AADDR.16	4.214	3.186	2.539	0.42	0.774	0.345	1.574	2	8.00	1.028
AADDR.14	4.203	2.744	2.539	0.42	0.774	0.345	1.564	2	7.42	1.459
AAADR.20	4.139	2.441	2.713	0.49	0.858	0.367	1.327	2	8.00	1.699
AAADR.19	4.123	2.470	2.713	0.49	0.858	0.367	1.310	2	7.42	1.654
AAADR.24	4.047	2.752	2.587	0.45	0.785	0.356	1.359	2	8.00	1.294
AAADR.23	4.038	2.565	2.587	0.45	0.785	0.356	1.351	2	7.42	1.474

**Table S7**. The distance between the pharmacophoric features							
Entry	Site1	Site2	Distance	Entry	Site1	Site2	Distance
ADDRR.4	A2	D3	3.596	ADDRR.4	D3	R7	7.726
ADDRR.4	A2	D4	3.225	ADDRR.4	D3	R8	5.55
ADDRR.4	A2	R7	5.446	ADDRR.4	D4	R7	5.942
ADDRR.4	A2	R8	3.707	ADDRR.4	D4	R8	3.754
ADDRR.4	D3	D4	2.026	ADDRR.4	R7	R8	2.193

**Table S8**. The angle between the pharmacophoric features									
Entry	Site1	Site2	Site3	Angle	Entry	Site1	Site2	Site3	Angle
ADDRR.4	D3	A2	D4	34	ADDRR.4	D3	D4	R7	147.4
ADDRR.4	D3	A2	R7	115.9	ADDRR.4	D3	D4	R8	146
ADDRR.4	D3	A2	R8	98.9	ADDRR.4	R7	D4	R8	1.7
ADDRR.4	D4	A2	R7	82.2	ADDRR.4	A2	R7	D3	24.8
ADDRR.4	D4	A2	R8	65.1	ADDRR.4	A2	R7	D4	32.5
ADDRR.4	R7	A2	R8	17.1	ADDRR.4	A2	R7	R8	29.8
ADDRR.4	A2	D3	D4	62.9	ADDRR.4	D3	R7	D4	8.1
ADDRR.4	A2	D3	R7	39.4	ADDRR.4	D3	R7	R8	6
ADDRR.4	A2	D3	R8	41.3	ADDRR.4	D4	R7	R8	3
ADDRR.4	D4	D3	R7	24.5	ADDRR.4	A2	R8	D3	39.8
ADDRR.4	D4	D3	R8	22.2	ADDRR.4	A2	R8	D4	51.2
ADDRR.4	R7	D3	R8	2.4	ADDRR.4	A2	R8	R7	133.1
ADDRR.4	A2	D4	D3	83.1	ADDRR.4	D3	R8	D4	11.8
ADDRR.4	A2	D4	R7	65.3	ADDRR.4	D3	R8	R7	171.6
ADDRR.4	A2	D4	R8	63.7	ADDRR.4	D4	R8	R7	175.3

Series -A

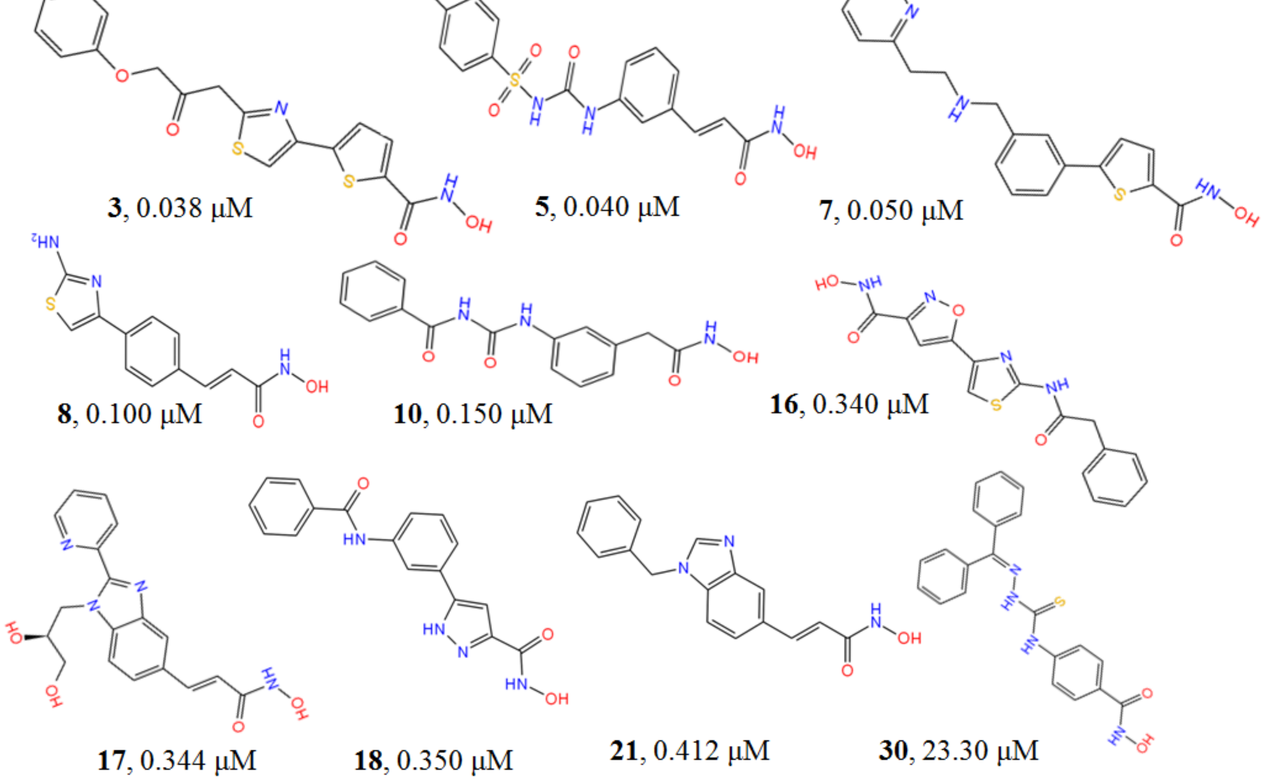

3, 0.038 µM; **5**, 0.040 µM; **7**, 0.050 µM; **8**, 0.100 µM; **10**, 0.150 µM; **16**, 0.340 µM; **17**, 0.344 µM; **18**, 0.350 µM; **21**, 0.412 µM; **30**, 23.30 µM

Series -B

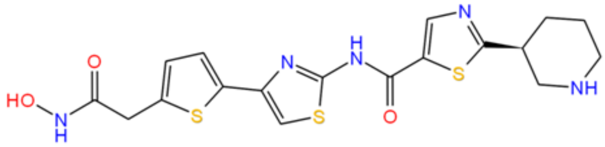

4, 0.040 µM

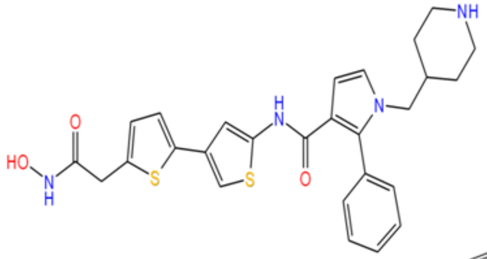

9, 0.140 µM

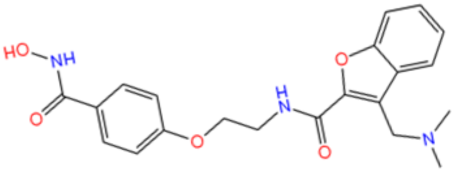

12, 0.190 µM

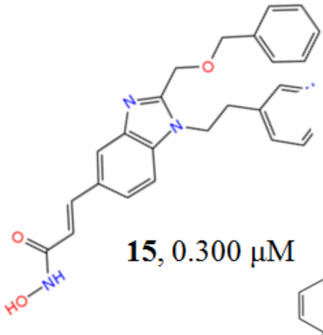

15, 0.300 µM

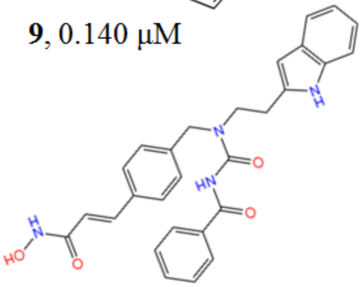

13, 0.210 µM

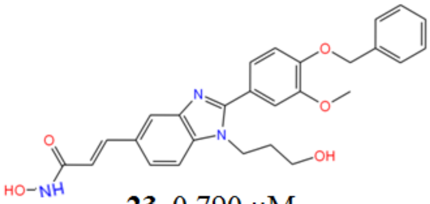

23, 0.790 µM

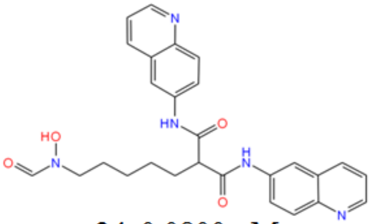

24, 0.0800 µM

Series-C

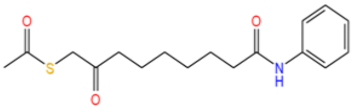

11, 0.190 µM

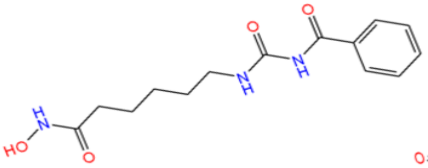

25, 1.070 µM

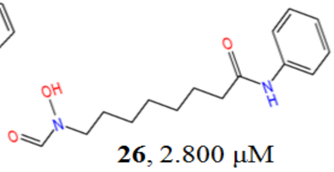

26, 2.800 µM

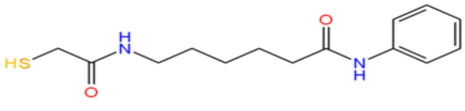

27, 3.890 µM

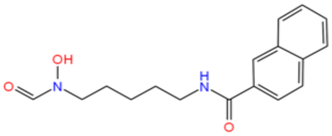

28, 7.000 µM

Series -D

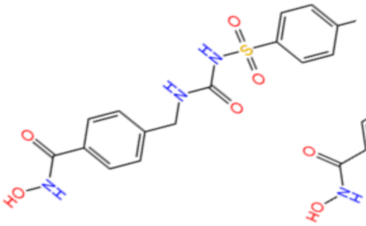

6, 0.049 µM

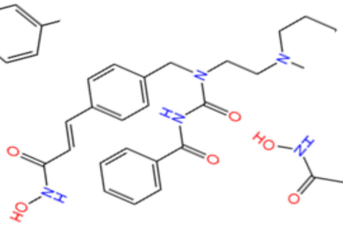

14, 0.230 µM

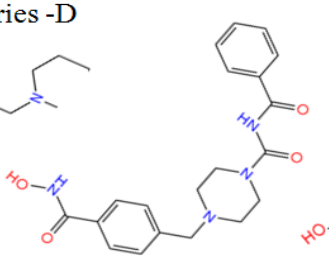

22, 0.460 µM

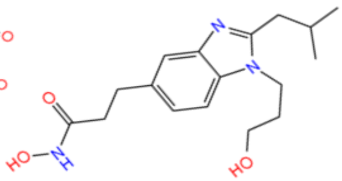

29, 0.230 µM

Series -E

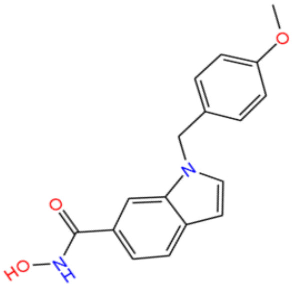

1, 0.01 μM

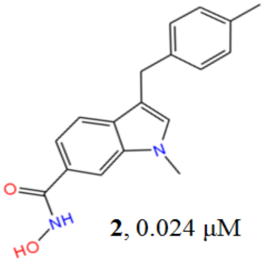

2, 0.024 μM

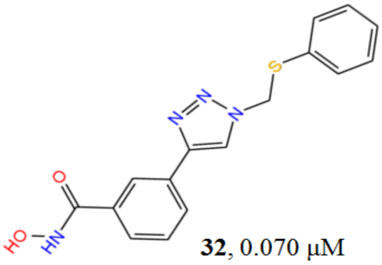

32, 0.070 μM

Series-F (three different structures)

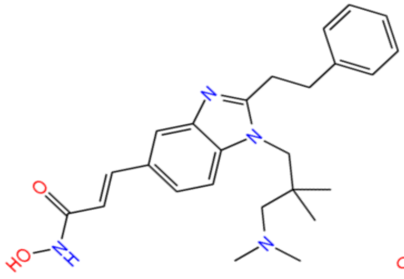

19, 0.353 μM

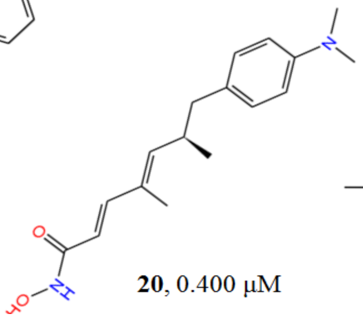

20, 0.400 μM

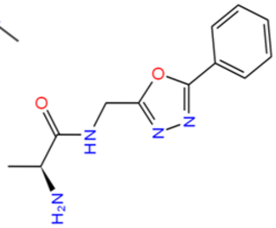

31, 23.300 μM

Figure S1. The structural classification of 32 selective known HDAC8 inhibitors A–F, used for model generation

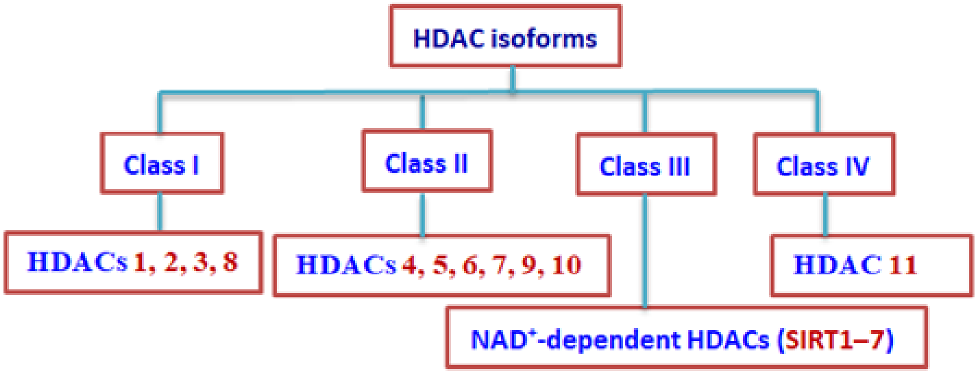

Figure S2. Classification of HDAC isoforms

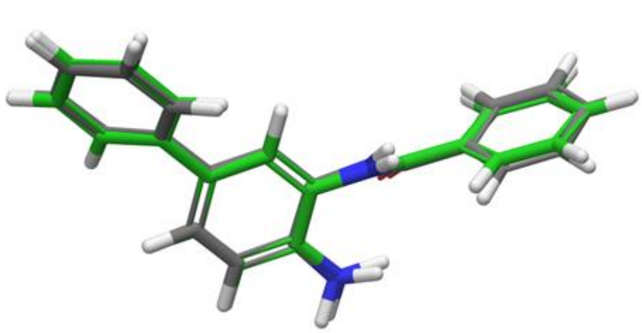

HDAC2, PDB ID: 3MAX [38], RMSD: 0.2067

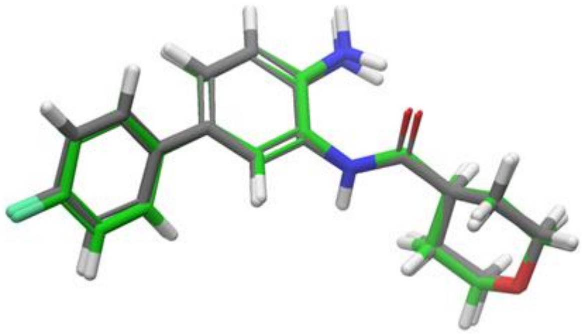

HDAC2, PDB ID:5IWG [57], RMSD: 0.2204

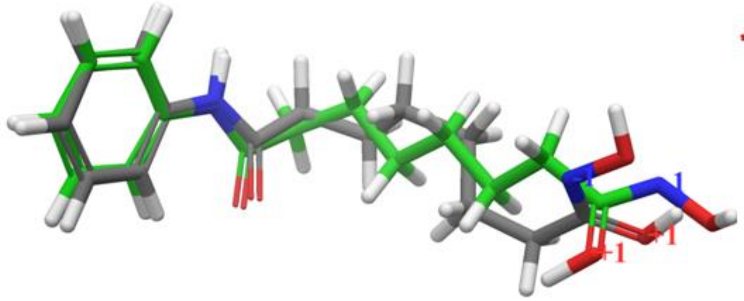

HDAC2, PDB ID:4LXZ [58] RMSD:1.5526

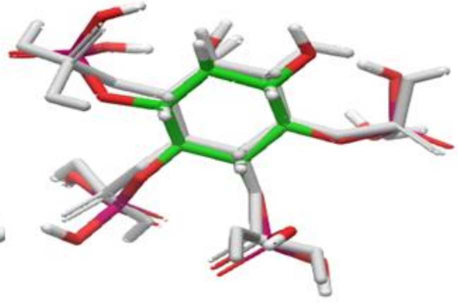

HDAC3, PDB ID: 4A69 [39] RMSD: 1.4762

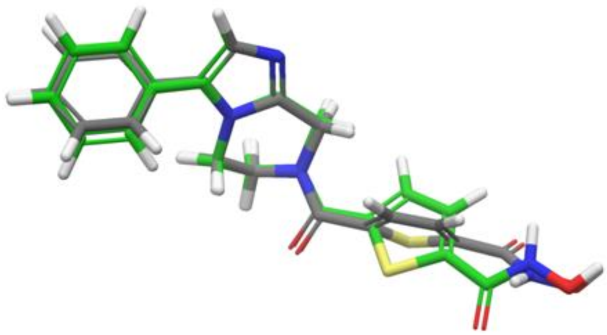

HDAC4, PDB ID: 2VQM [40], RMSD: 1.5703

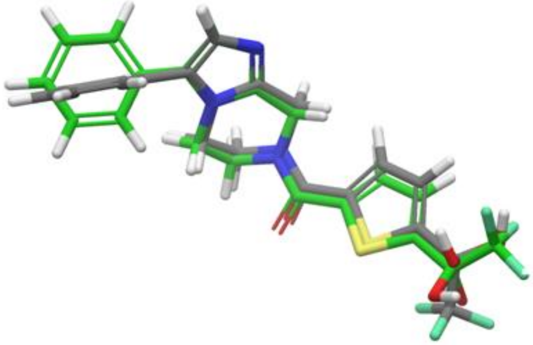

HDAC4, PDB ID: 2VQO [59], RMSD:1.6445

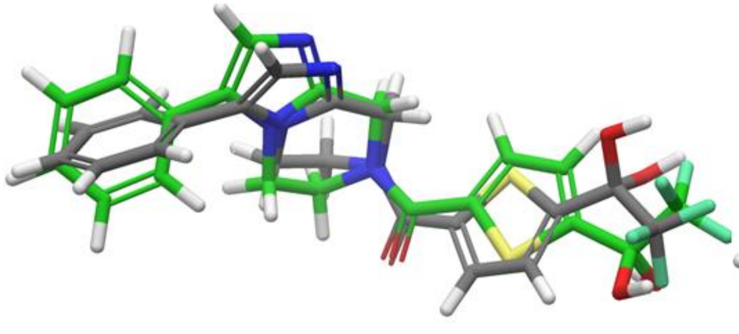

HDAC4, PDB ID: 2VQJ [59], RMSD: 1.8655

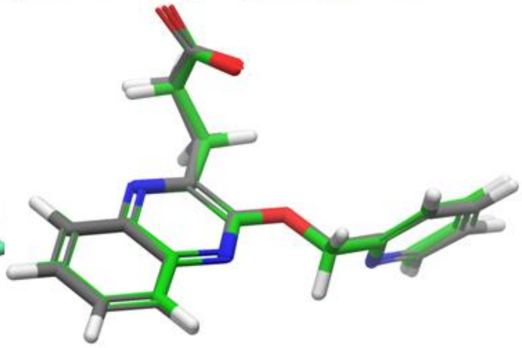

HDAC6, PDB ID: 5WPB [41], RMSD: 0.2874

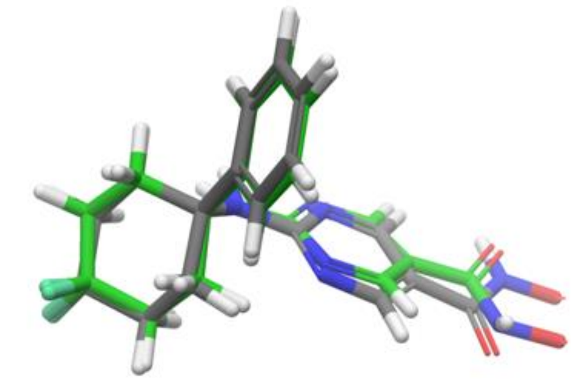

HDAC6, PDB ID: 5WGM [60], RMSD: 0.8484

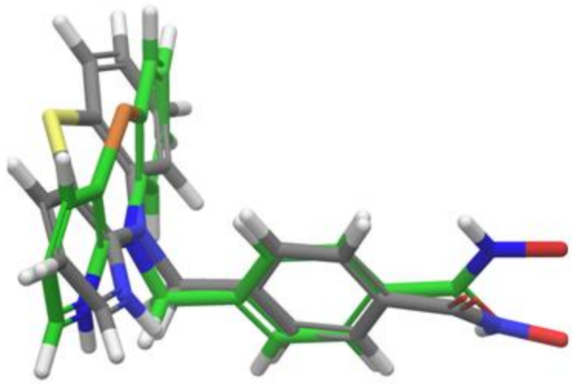

HDAC6, PDB ID: 5W5K [61], RMSD:1.1551

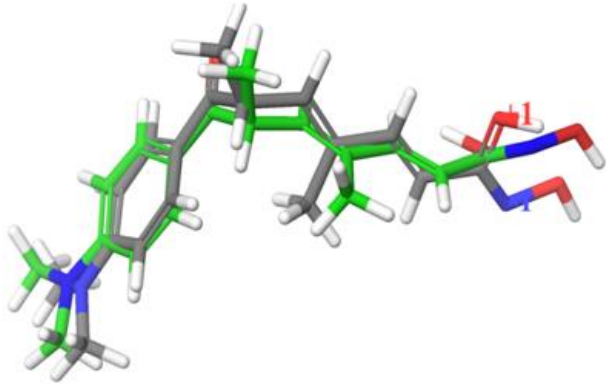

HDAC6, PDB ID: 5WGI [60], RMSD:1.1551

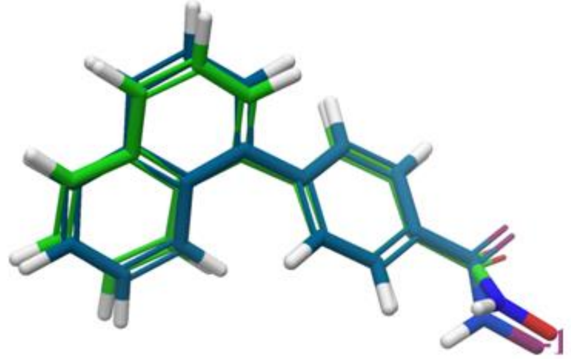

HDAC8, PDB ID: 5FCW [62], RMSD:0.4290

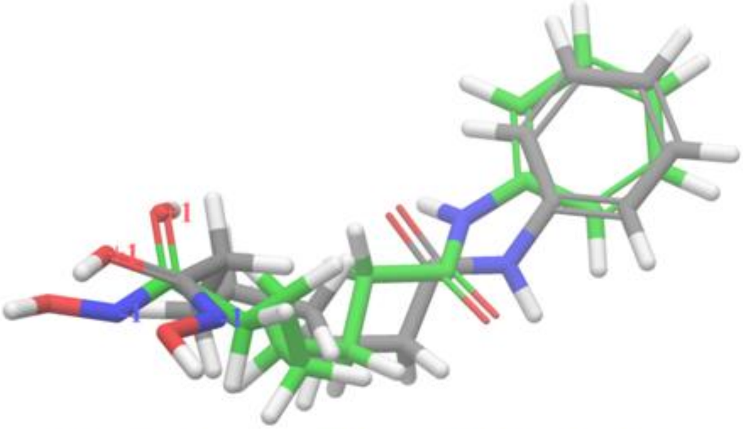

HDAC8, PDB ID: 1T69 [63], RMSD:1.9849

Figure S3. Superposition of the docked co-ligands (ash coloured) on their respective crystallographic bound conformation (green) of different HDACs (HDAC2, HDAC3, HDAC4, HDAC6, HDAC8).

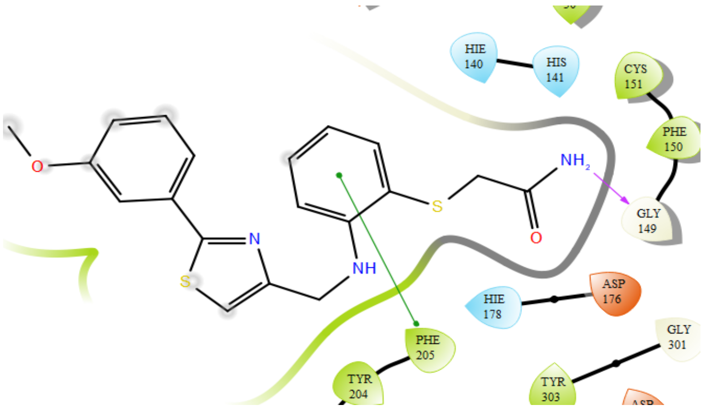

SD-01, XPGS: –6.8

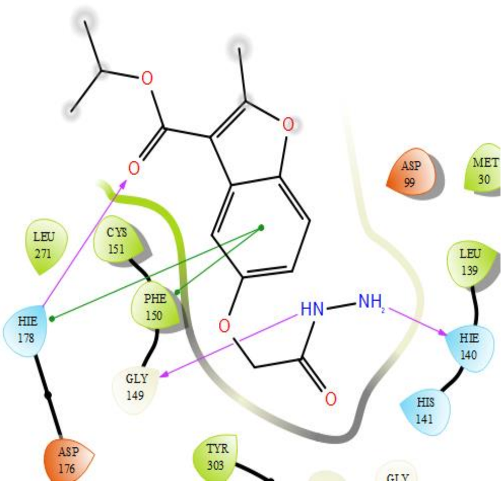

SD-02, XPGS: –7.7

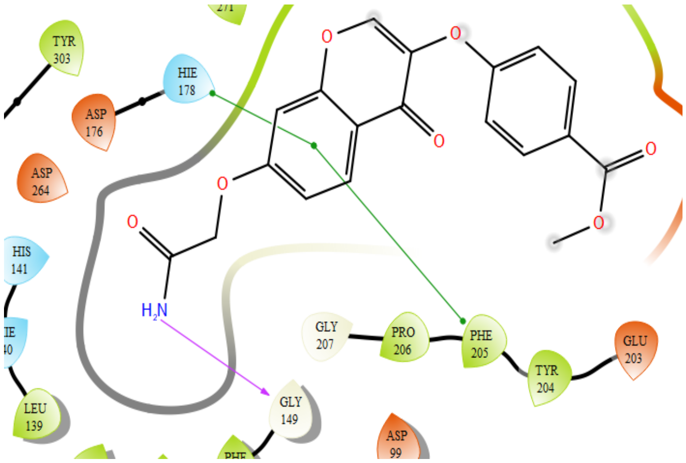

SD-03, XPGS: –7.6

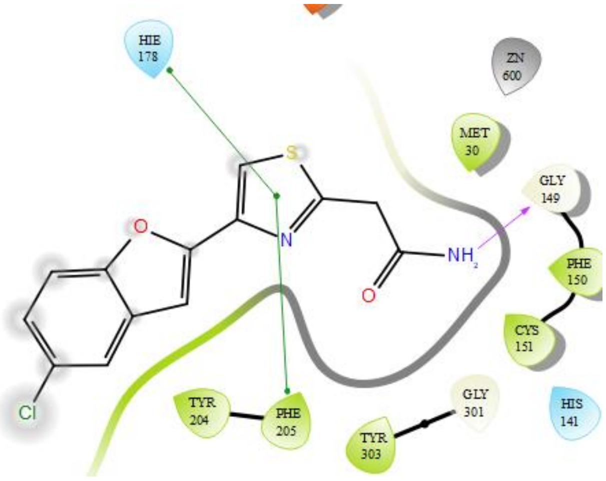

SD-04, XPGS: –4.2

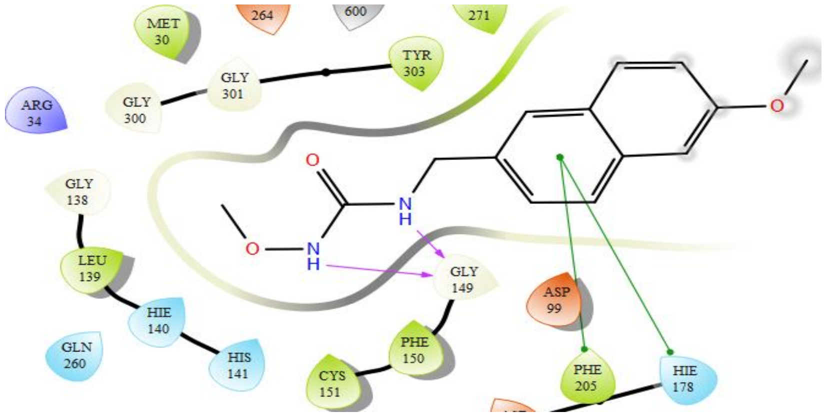

SD-05, XPGS: –8.2

Figure S4a. 2D-ligand interaction diagram of selected inhibitors SD-01, SD-02, SD-03, SD-04 and SD-05 with HDAC1 (XPGS: XP Glide Score)

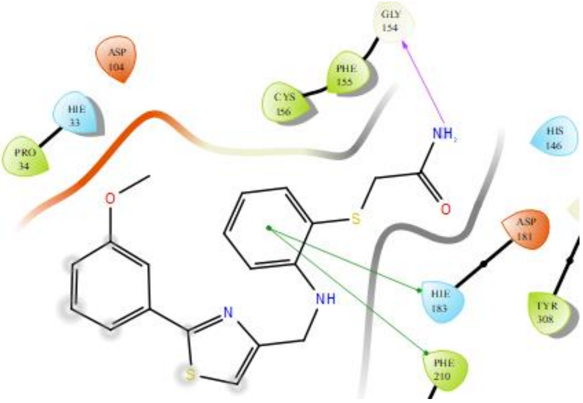

SD-01, XPGS: –7.6

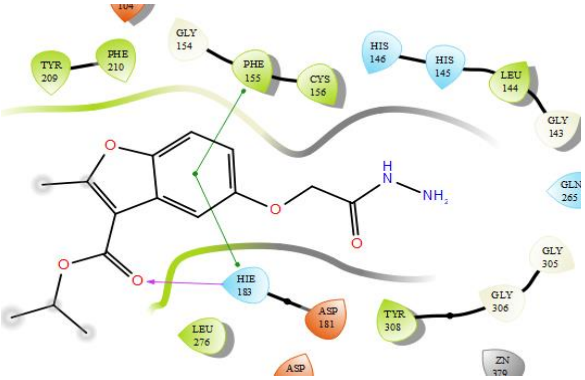

SD-02, XPGS: –8.7

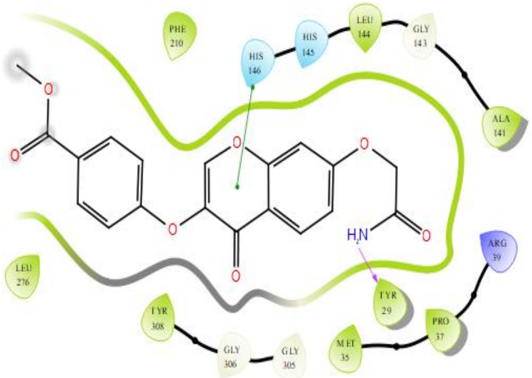

SD-03, XPGS: –9.5

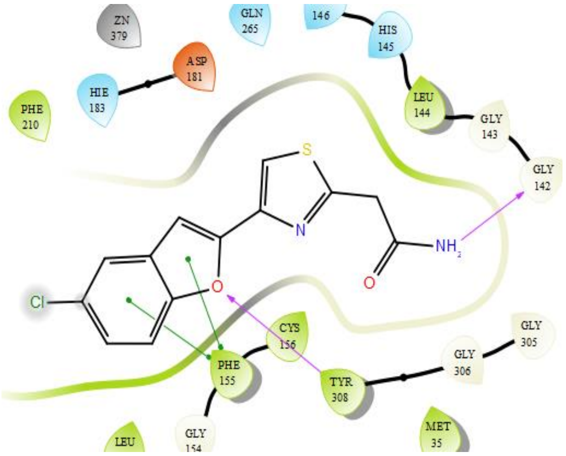

SD-04, XPGS: –6.3

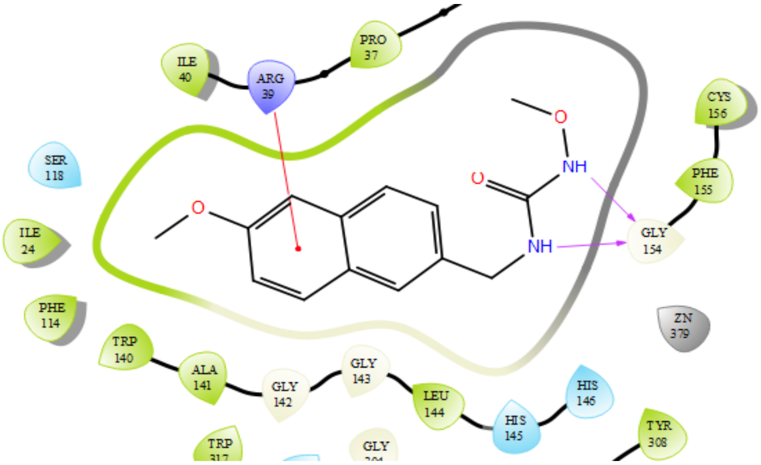

SD-05, XPGS: –8.4

Figure S4b. 2D-ligand interaction diagram of selected inhibitors SD-01, SD-02, SD-03, SD-04 and SD-05 with HDAC2

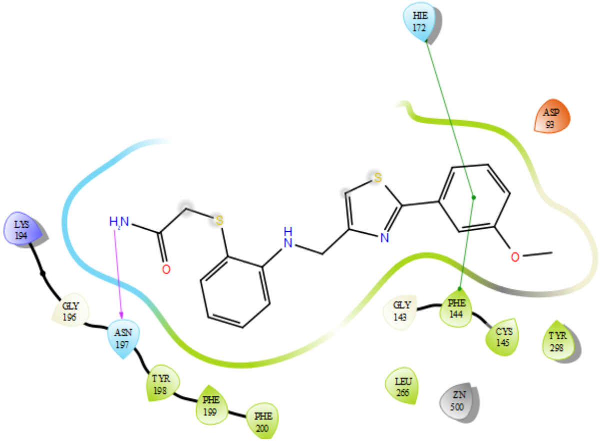

SD-01, XPGS: –5.5

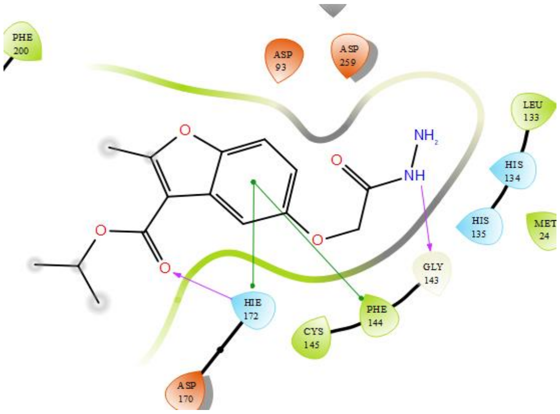

SD-02, XPGS: –8.2

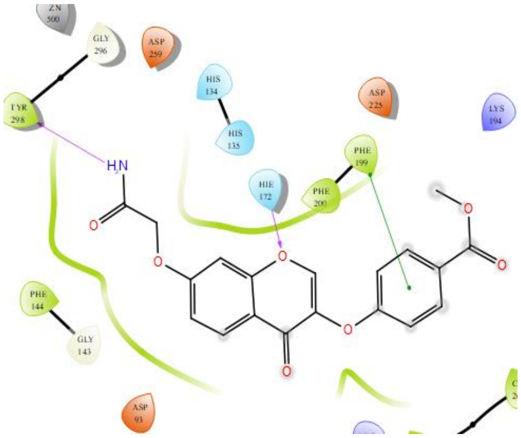

SD-03, XPGS: –5.6

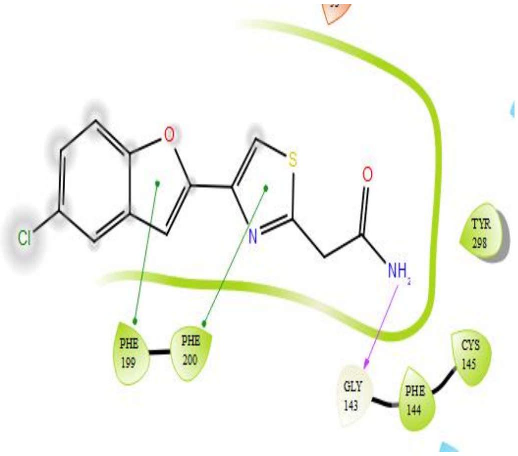

SD-04, XPGS: –4.2

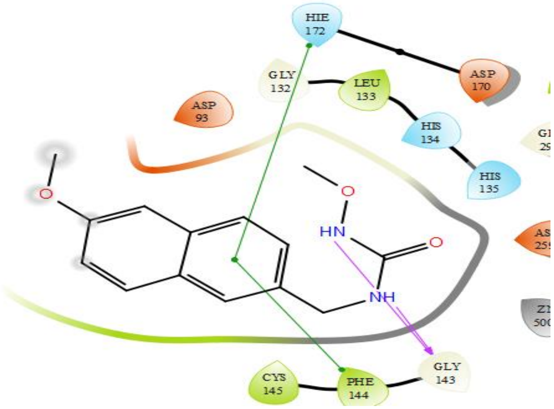

SD-05, XPGS: –8.5

Figure S4c. 2D-ligand interaction diagram of selected inhibitors SD-01, SD-02, SD-03, SD-04 and SD-05 with HDAC3

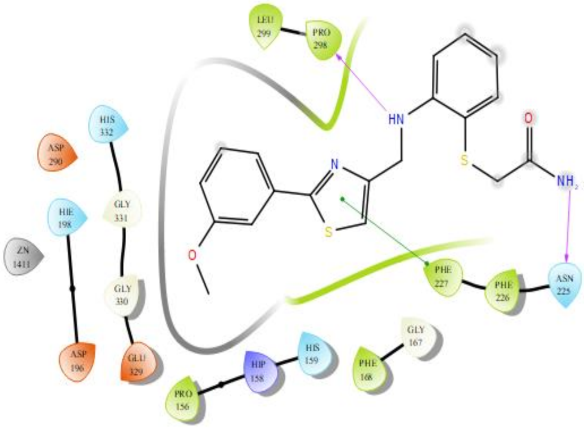

SD-01, XPGS: –7.3

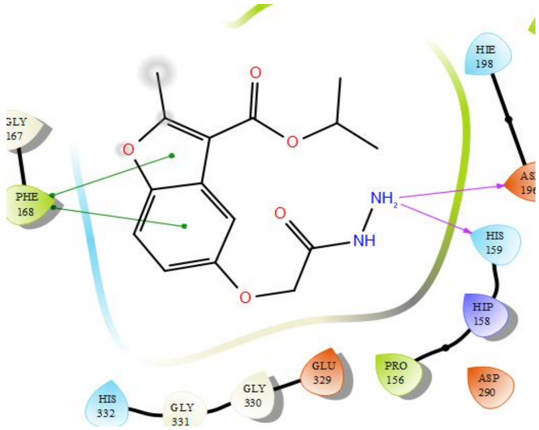

SD-02, XPGS: –7.32

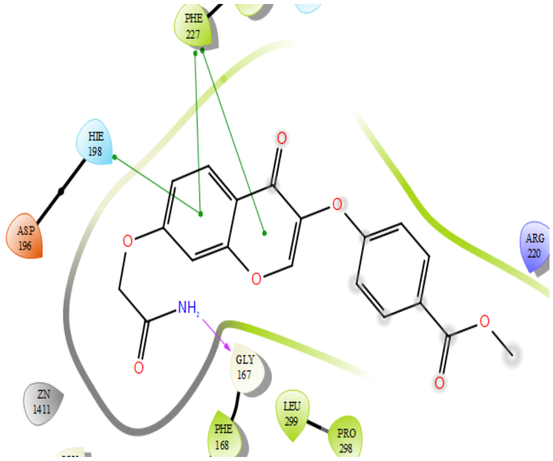

SD-03, XPGS: –7.3

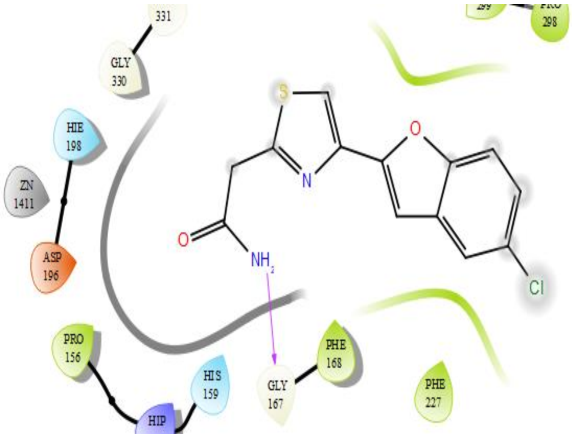

SD-4, XPGS: –6.7

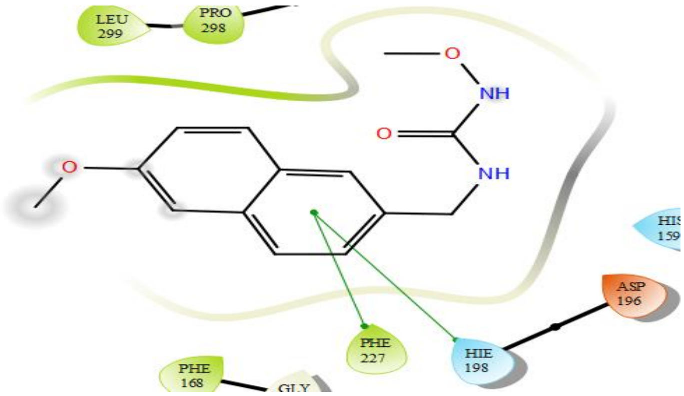

SD-05, XPGS: –6.8

Figure S4d. 2D-ligand interaction diagram of selected inhibitors SD-01, SD-02, SD-03, SD-04 and SD-05 with HDAC4

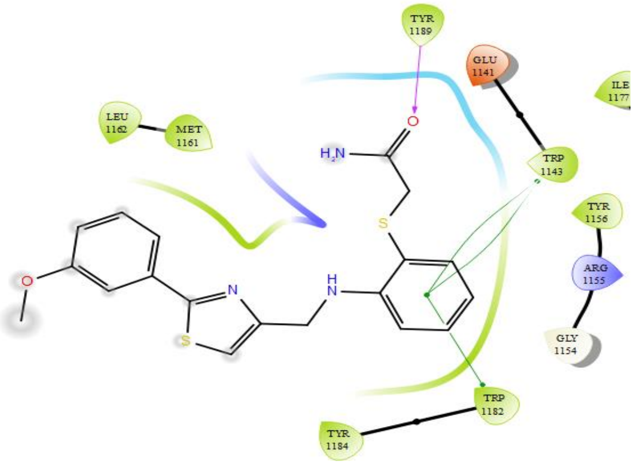

SD-01, XPGS: –4.5

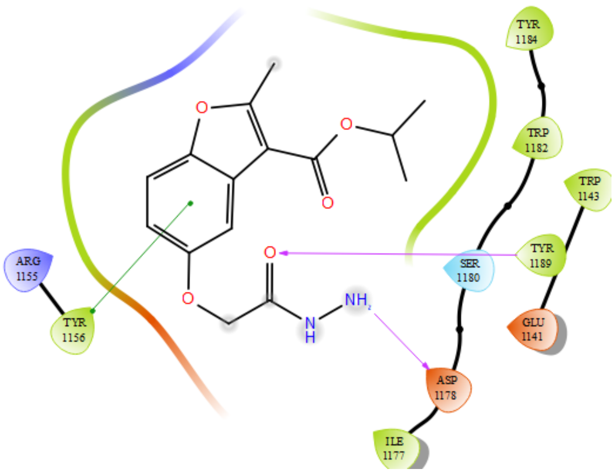

SD-02, XPGS: –5.6

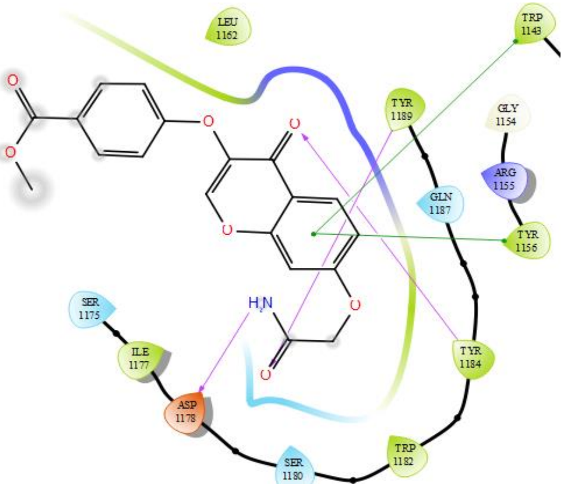

SD-03, XPGS: –5.2

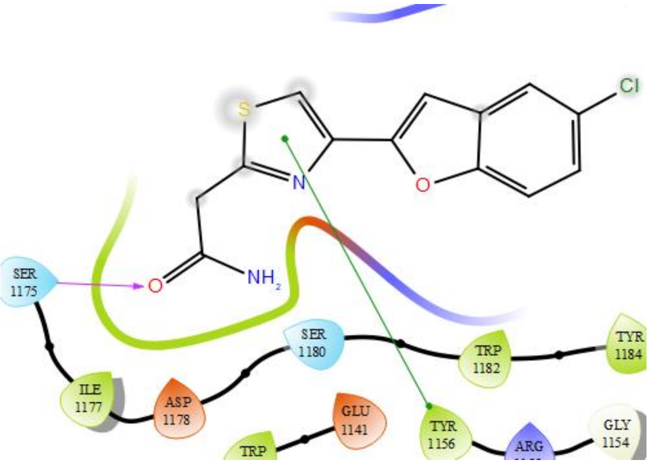

SD-04, XPGS: –5.3

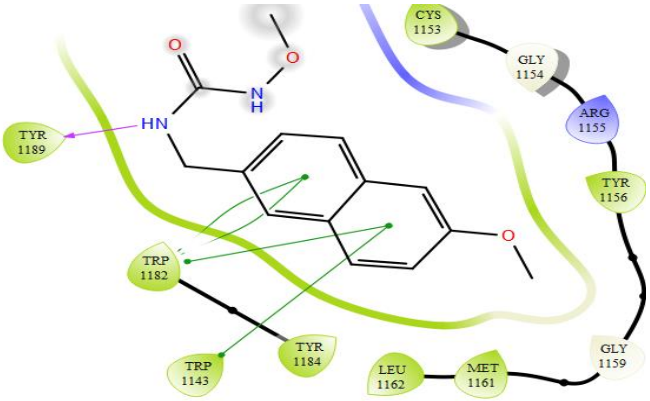

SD-05, XPGS: –5.5

Figure S4e. 2D-ligand interaction diagram of selected inhibitors SD-01, SD-02, SD-03, SD-04 and SD-05 with HDAC6 (5WPB)

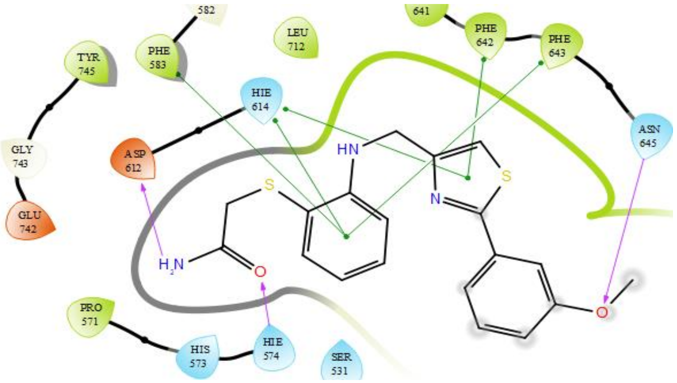

SD-01, XPGS: –8.0

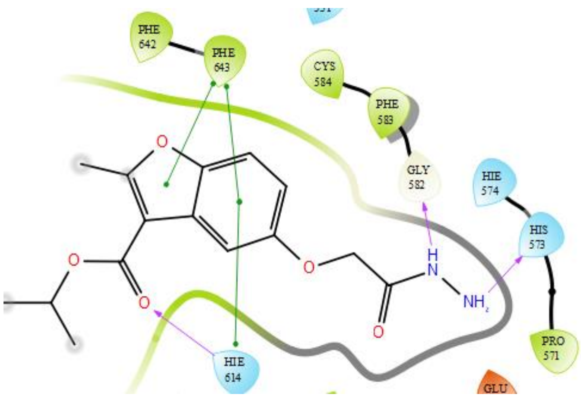

SD-02, XPGS: –9.2

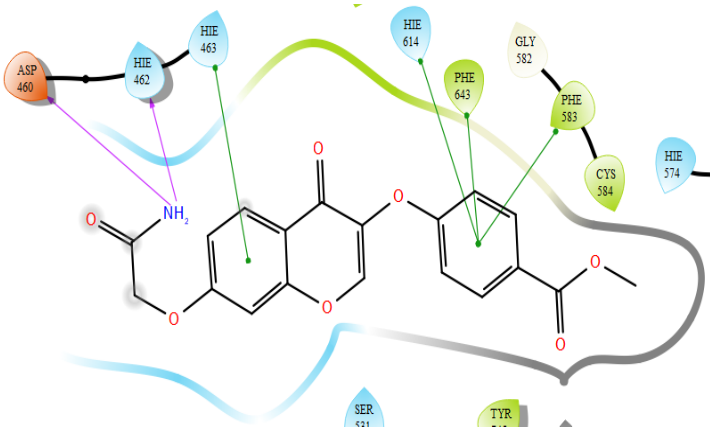

SD-03, XPGS: –8.5

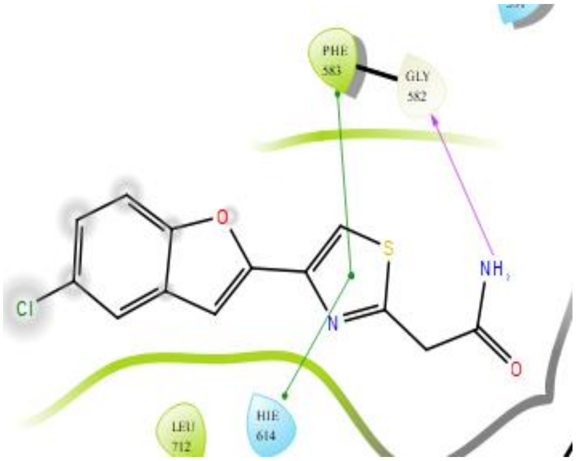

SD-04, XPGS: –7.6

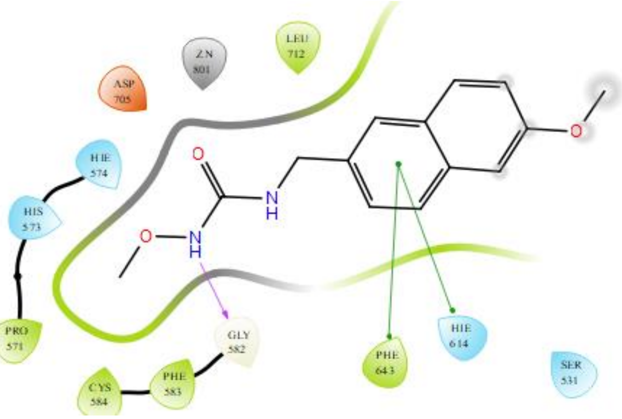

SD-05, XPGS: –8.0

Figure S4f. 2D-ligand interaction diagram of selected inhibitors SD-01, SD-02, SD-03, SD-04 and SD-05 with HDAC6 (PDB ID: 5WGI)

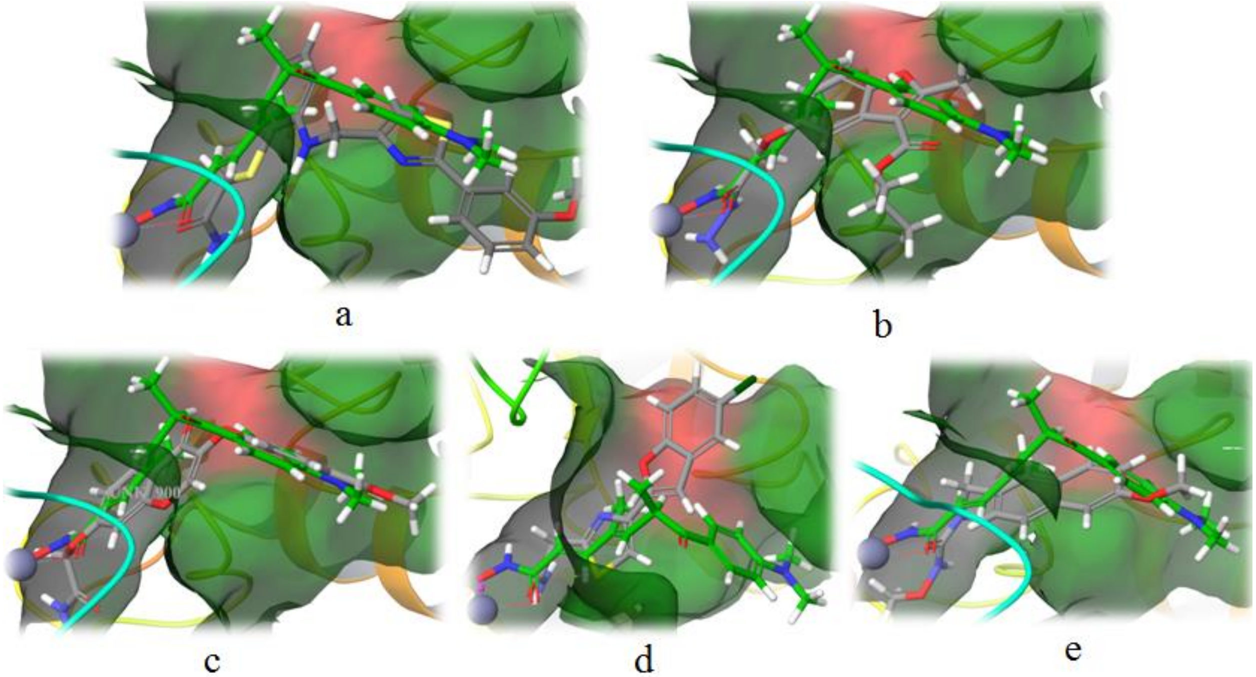

Figure S5. Visualization of binding mode of SD-01 (a), SD-02 (b), SD-03 (c), SD-04 (d), and SD-05 (e) by superposition on crystallographic bound co-ligand TSN of HDAC8 (PDB ID: 1T64) in its active site

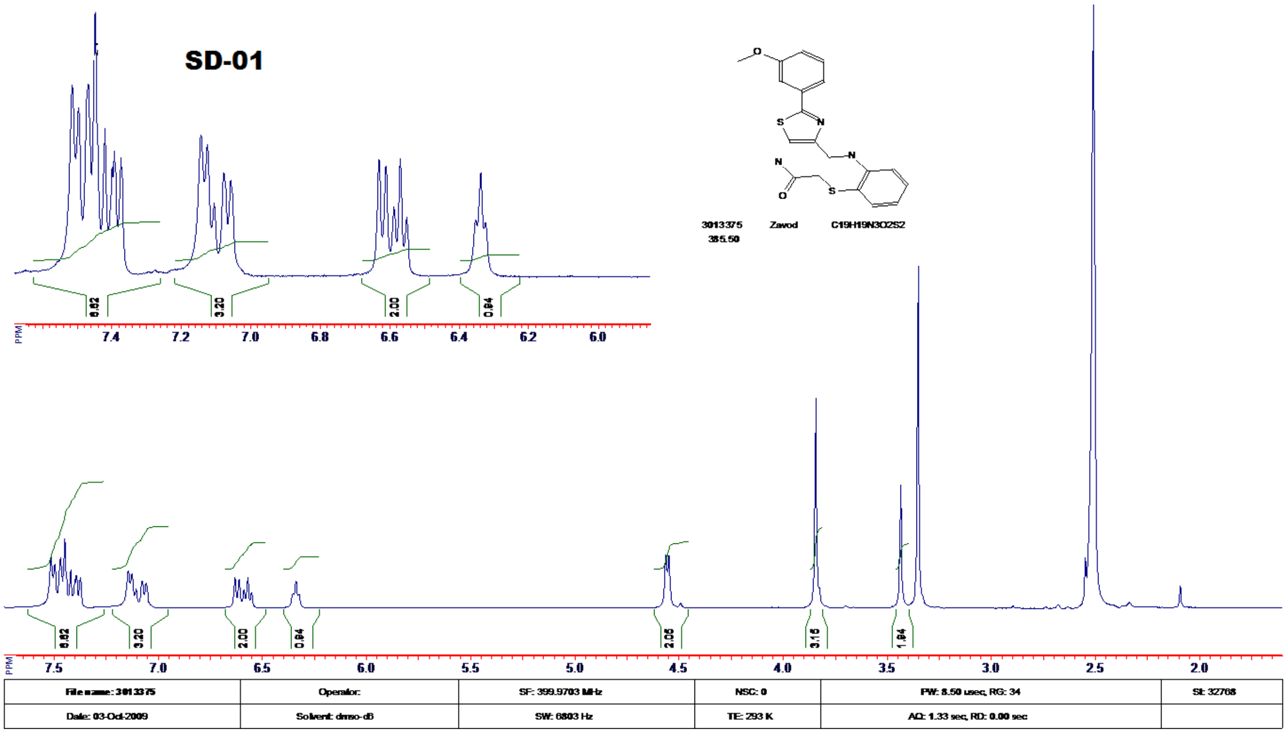

Figure S6a. ^{1}H-NMR of compounds **SD-01**

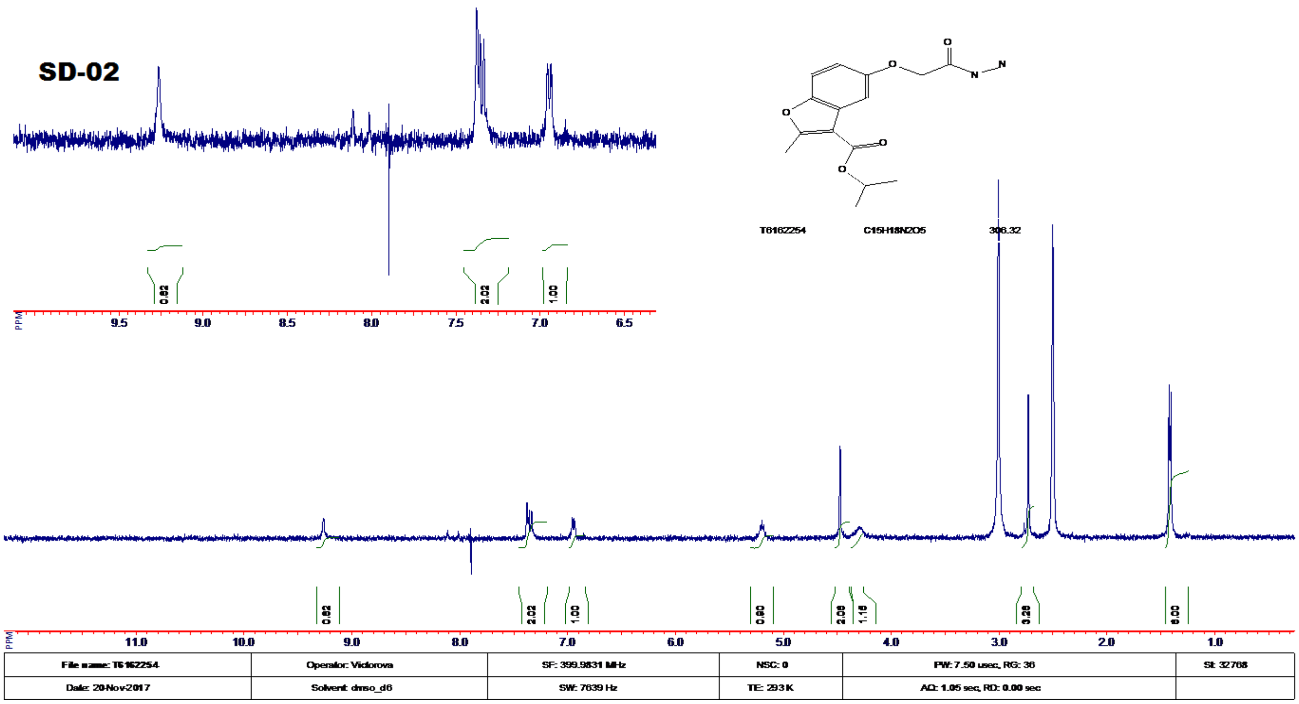

Figure S6b. ^{1}H-NMR of compounds SD-02

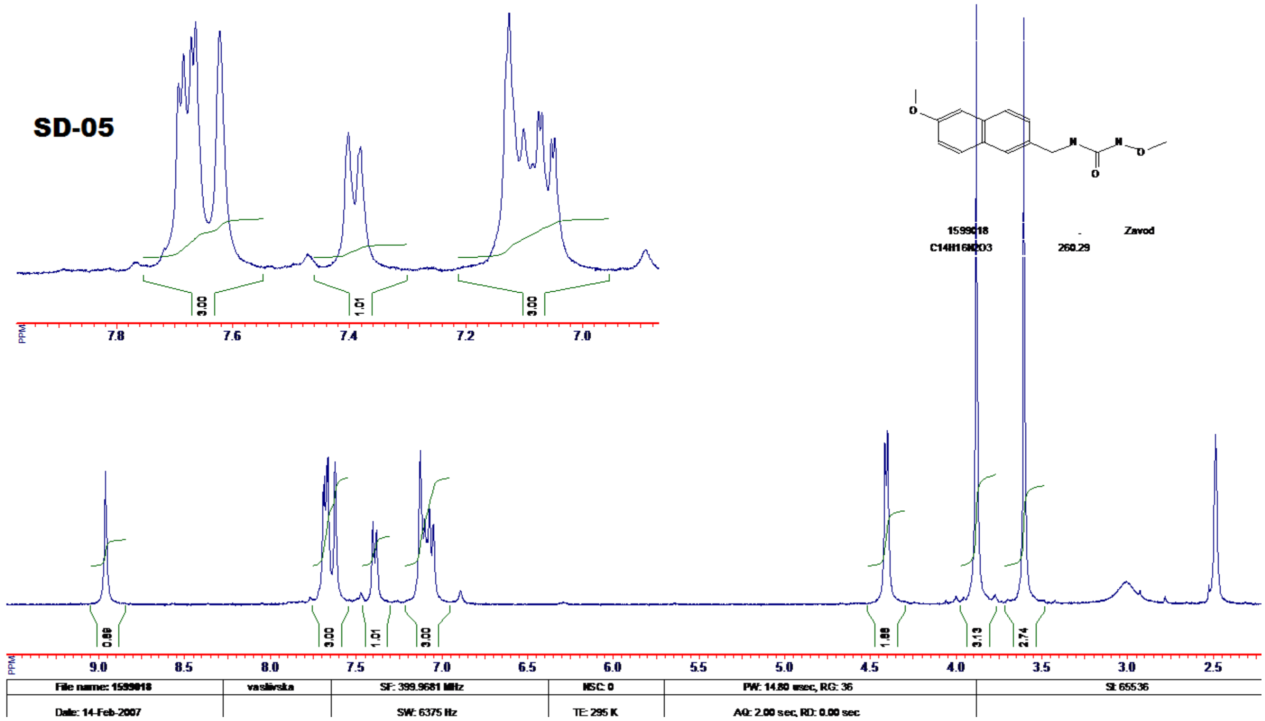

Figure S6c. ^{1}H-NMR of compounds SD-05

SD-01

HPLC Report

Sample Information

Sample Name : PM-SD-01-2-MG
Date Acquired : 12/24/2018 3:42:41 PM
Tray# : 1
Vial# : 76
Injection Volume : 5
Data File : 241218.2.lcd
Method File : CD HPLC.lcm
Report Format File : LC-MS Data Report.lsr
Comment : Chromatographic Conditions :
Colum: X-SELECT CSH (150 X 4.6mm, 5.0u)
Mobile Phase:GRADIENT-M
Processed by : System Administrator
Date Processed : 12/24/2018 4:12:45 PM

Chromatogram
D:\DATA\SCS-18\241218.2.lcd

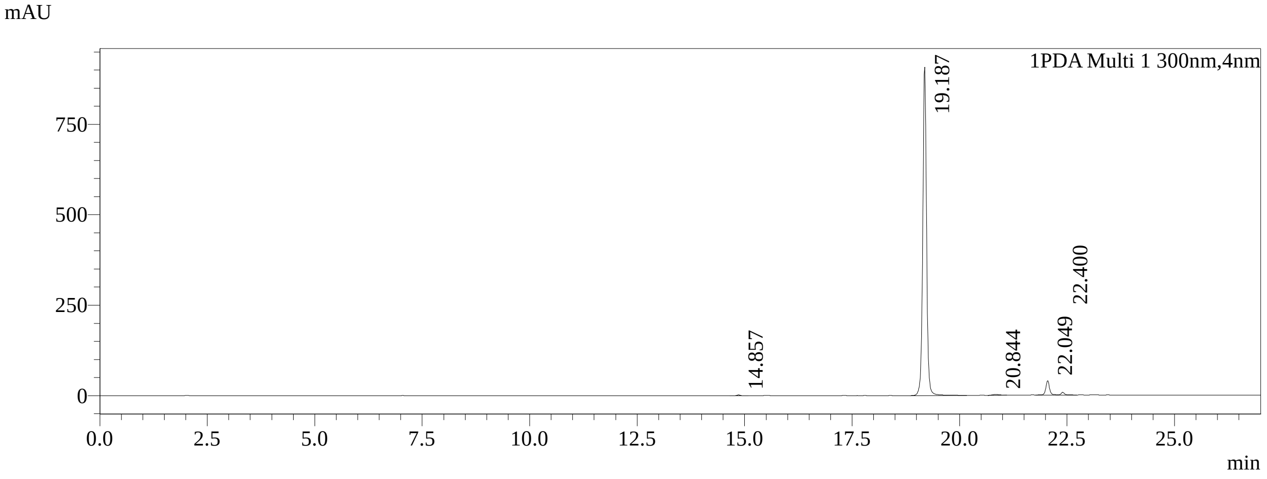

Peak Table

PDA Ch1 300nm

Peak#	Ret. Time	Peak Start	Peak End	Area	Area%	Height%
1	14.857	14.656	15.083	16803	0.285	0.281
2	19.187	18.848	20.160	5579291	94.520	94.543
3	20.844	20.661	21.088	23065	0.391	0.236
4	22.049	21.771	22.283	238797	4.046	4.147
5	22.400	22.283	22.720	44802	0.759	0.793
Total				5902759	100.000	100.000

Figure S7a. HPLC purity of compound SD-01

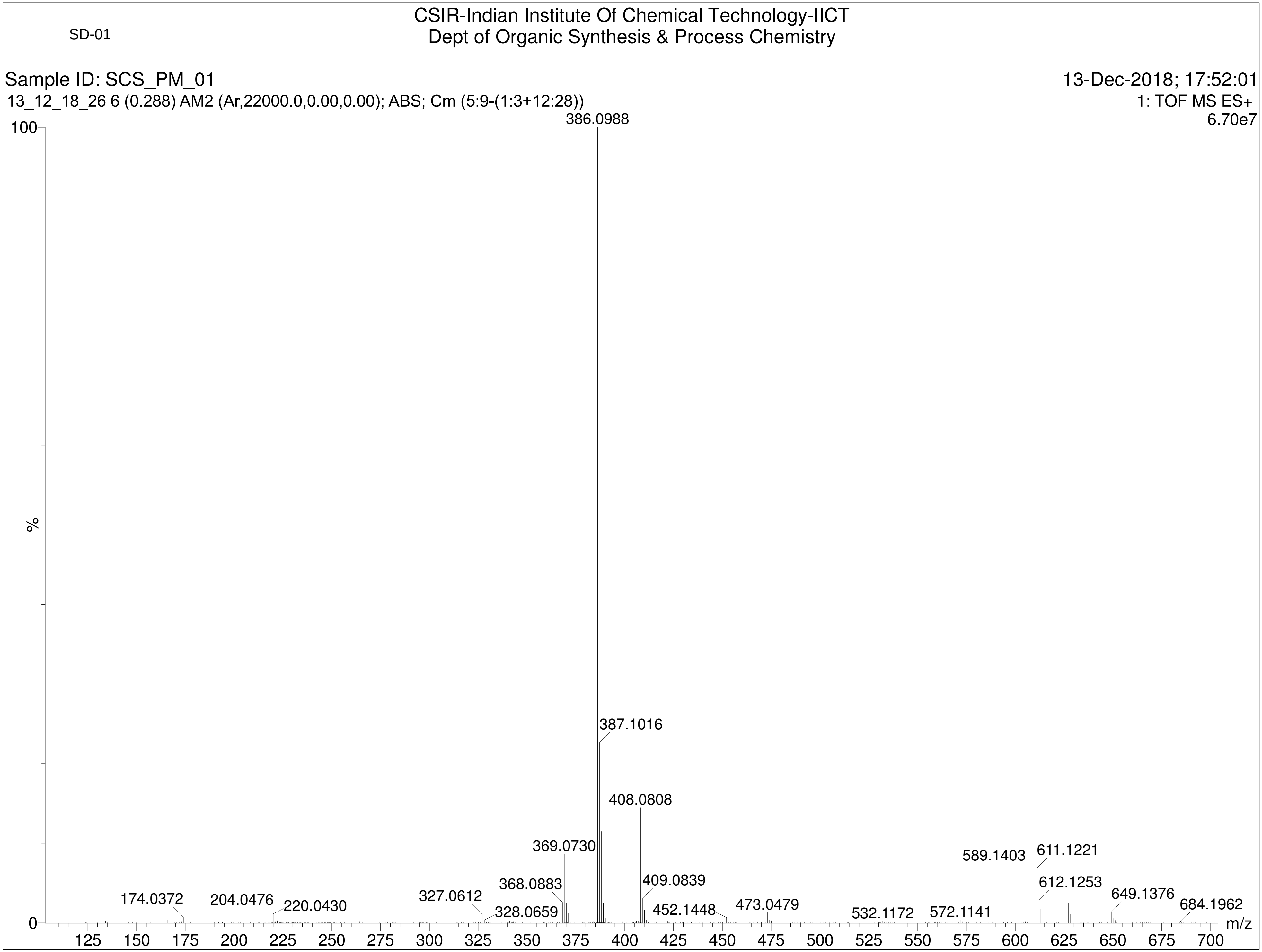

Figure S8a. Mass spectra of compound SD-01

SD-02

HPLC Report

Sample Information

Sample Name : PM-SD-02-2-MG
Date Acquired : 12/24/2018 4:14:25 PM
Tray# : 1
Vial# : 77
Injection Volume : 5
Data File : 241218.3.lcd
Method File : CD HPLC.lcm
Report Format File : LC-MS Data Report.lsr
Comment : Chromatographic Conditions :
Colum: X-SELECT CSH (150 X 4.6mm, 5.0u)
Mobile Phase:GRADIENT-M
Processed by : System Administrator
Date Processed : 12/24/2018 4:44:29 PM

Chromatogram
D:\DATA\SCS-18\241218.3.lcd

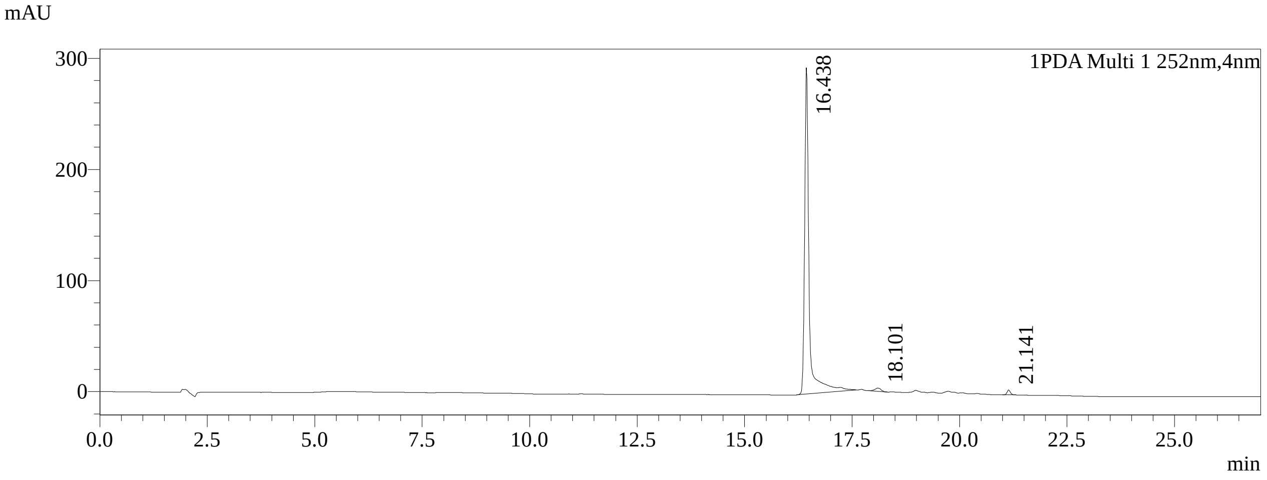

Peak Table

PDA Ch1 252nm

Peak#	Ret. Time	Peak Start	Peak End	Area	Area%	Height%
1	16.438	16.213	17.579	2113363	97.630	97.594
2	18.101	17.867	18.347	28200	1.303	0.942
3	21.141	21.003	21.301	23094	1.067	1.464
Total				2164657	100.000	100.000

Figure S7b. HPLC purity of compound SD-02

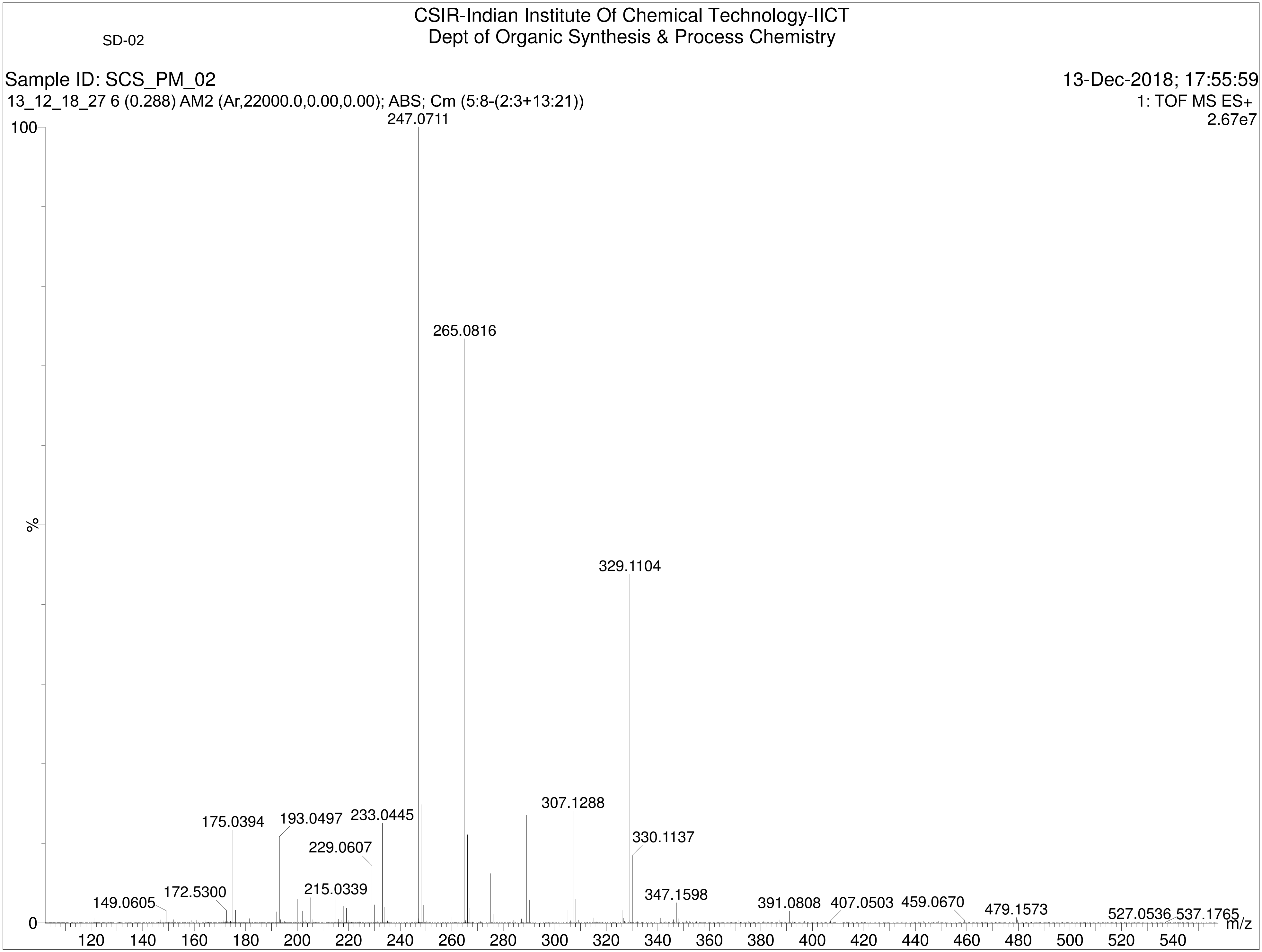

Figure S8b. Mass spectra of compound SD-02

SHIMADZU LabSolutions

LC-MS DATA REPORT

SD-03

Sample Code : PM-SD-03-2MG
Data File : 241218.5.lcd
Method : CD HPLC.lcm
Injection Volume : 3
Date Acquired : 12/24/2018 8:06:16 PM
Report File : LC-MS Data Report.lsr
Chromatographic Conditions : Chromatographic Conditions :
Colum: LUNA C8 (250 X 5.0mm), 4.6 um
Mobile Phase:GRADIENT
Flowrate : 1.0 mL/min

Chromatogram

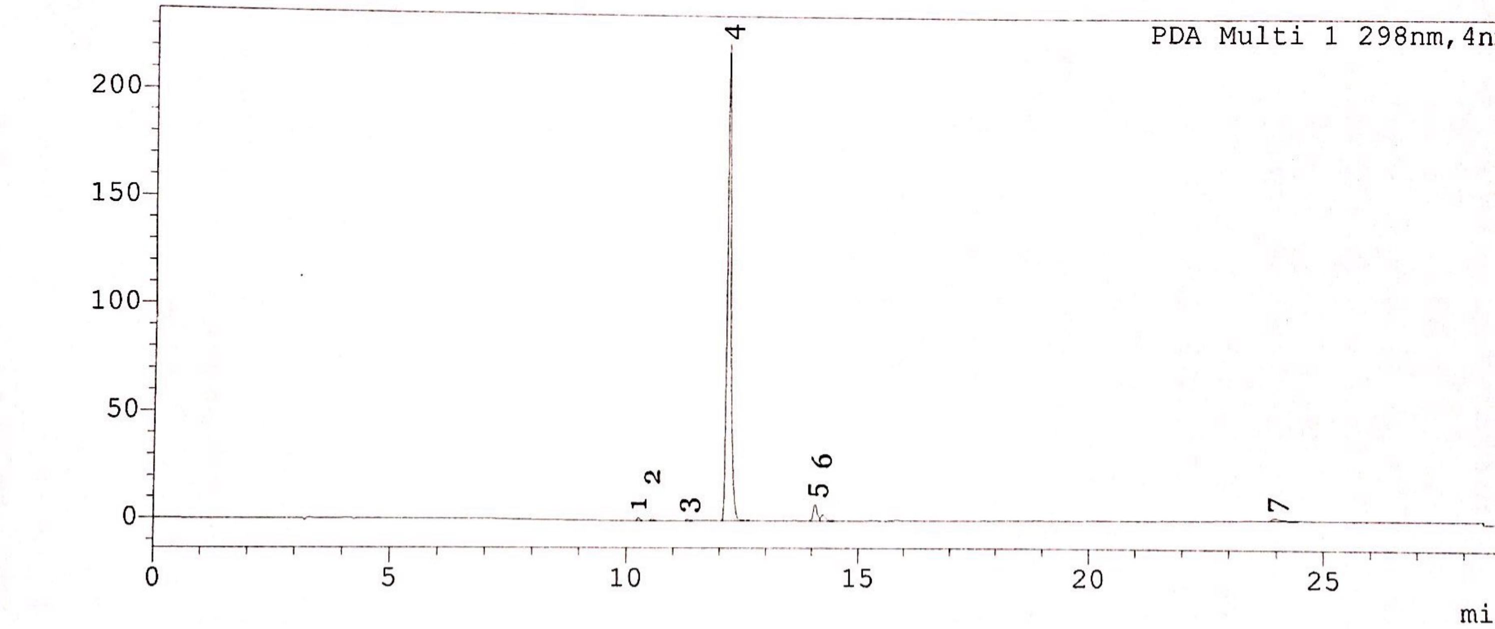

Peak Table

PDA Ch1 298nm

Peak#	Ret. Time	Peak Start	Peak End	Area	Area%
1	10.254	10.197	10.453	6133	0.443
2	10.545	10.453	10.720	3040	0.220
3	11.306	11.221	11.456	3600	0.260
4	12.169	11.936	12.768	1286907	93.014
5	14.070	13.803	14.176	47521	3.435
6	14.241	14.176	14.539	20290	1.466
7	23.994	23.829	24.469	16072	1.162
Total				1383564	100.000

Figure S7c. HPLC purity compound SD-03

Scanned with CamScanner

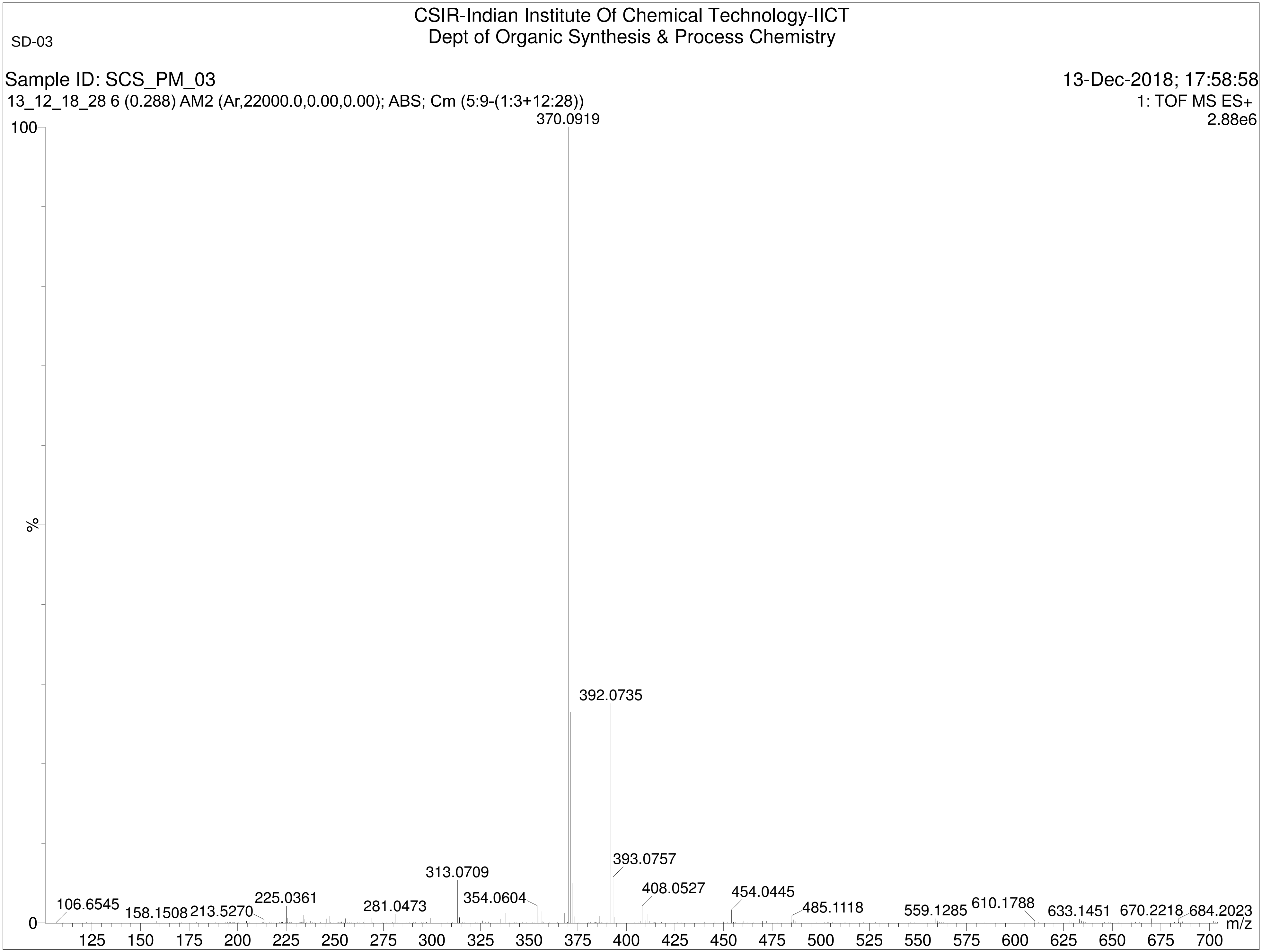

Figure S8c. Mass spectra of compound SD-03

SD-04

HPLC Report

Sample Information

Sample Name	: SCS-SD-04-292.739
Date Acquired	: 12/28/2018 4:03:51 PM
Tray#	: 1
Vial#	: 81
Injection Volume	: 5
Data File	: 281218.2.lcd
Method File	: CD HPLC.lcm
Report Format File	: LC-MS Data Report.lsr
Comment	: Chromatographic Conditions :<br>Colum: LUNA C18 (250 X 4.6mm, 5.0u)<br>Mobile Phase:GRADIENT
Processed by	: System Administrator
Date Processed	: 12/28/2018 4:33:54 PM

Chromatogram
D:\DATA\SCS-18\281218.2.lcd

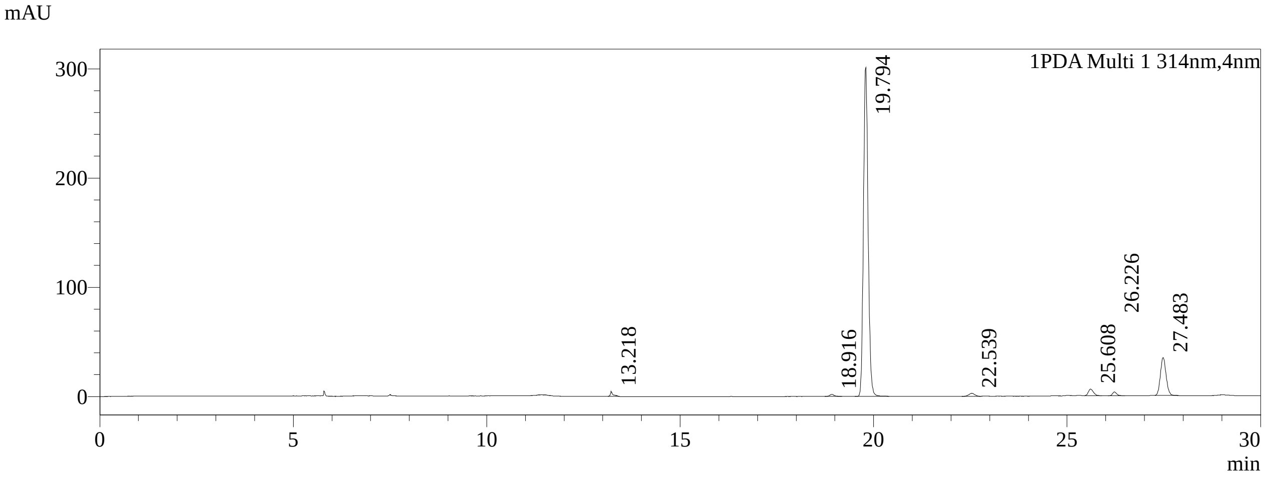

Peak Table

PDA Ch1 314nm

Peak#	Ret. Time	Peak Start	Peak End	Area	Area%	Height%
1	13.218	13.141	13.419	20660	0.684	1.270
2	18.916	18.741	19.168	15852	0.525	0.547
3	19.794	19.520	20.384	2522252	83.471	84.972
4	22.539	22.283	22.763	29893	0.989	0.785
5	25.608	25.451	25.888	53807	1.781	1.701
6	26.226	26.059	26.485	25309	0.838	0.962
7	27.483	27.275	27.840	353942	11.713	9.765
Total				3021715	100.000	100.000

Figure S7d. HPLC purity of compound SD-04

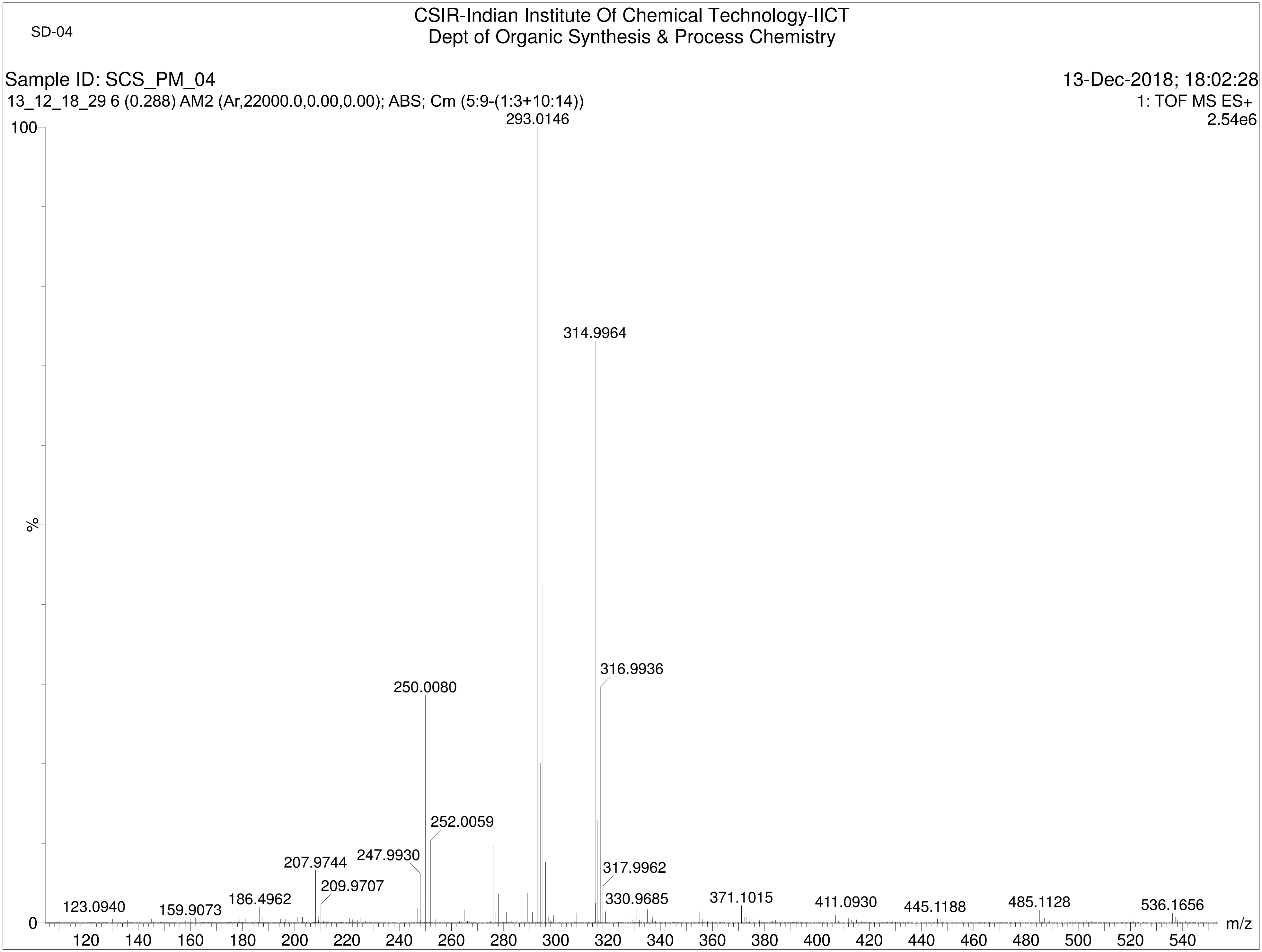

Figure S8d. Mass spectra of compound SD-04

SD-05

HPLC Report

Sample Information

Sample Name : PM-SD-05-2MG
Date Acquired : 12/24/2018 7:35:41 PM
Tray# : 1
Vial# : 78
Injection Volume : 3
Data File : 241218.4.lcd
Method File : CD HPLC.lcm
Report Format File : LC-MS Data Report.lsr
Comment : Chromatographic Conditions :
Colum: LUNA C8 (250 X 5.0mm), 4.6 um
Mobile Phase:GRADIENT
Flowrate : 1.0 mL/min
Processed by : System Administrator
Date Processed : 12/24/2018 8:05:43 PM

Chromatogram
D:\DATA\SCS-18\241218.4.lcd

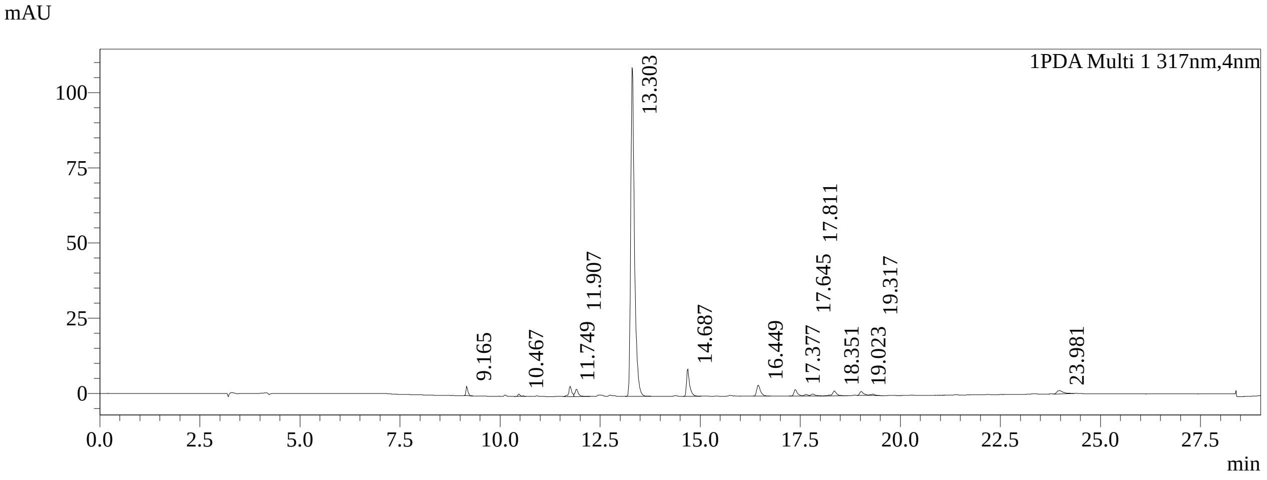

Peak Table

PDA Ch1 317nm

Peak#	Ret. Time	Peak Start	Peak End	Area	Area%	Height%
1	9.165	9.109	9.301	10180	1.146	2.169
2	10.467	10.411	10.635	3339	0.376	0.547
3	11.749	11.584	11.840	17636	1.986	2.418
4	11.907	11.840	12.245	14093	1.587	1.726
5	13.303	13.131	13.760	710768	80.034	78.515
6	14.687	14.581	15.029	48803	5.495	6.499
7	16.449	16.320	16.747	24341	2.741	2.621
8	17.377	17.216	17.557	12890	1.451	1.498
9	17.645	17.557	17.728	3163	0.356	0.362
10	17.811	17.728	18.069	4743	0.534	0.462
11	18.351	18.069	18.688	13150	1.481	1.153
12	19.023	18.923	19.200	9590	1.080	0.944
13	19.317	19.200	19.552	3821	0.430	0.341
14	23.981	23.840	24.331	11563	1.302	0.744
Total				888079	100.000	100.000

Figure S7e. HPLC purity compound SD-05

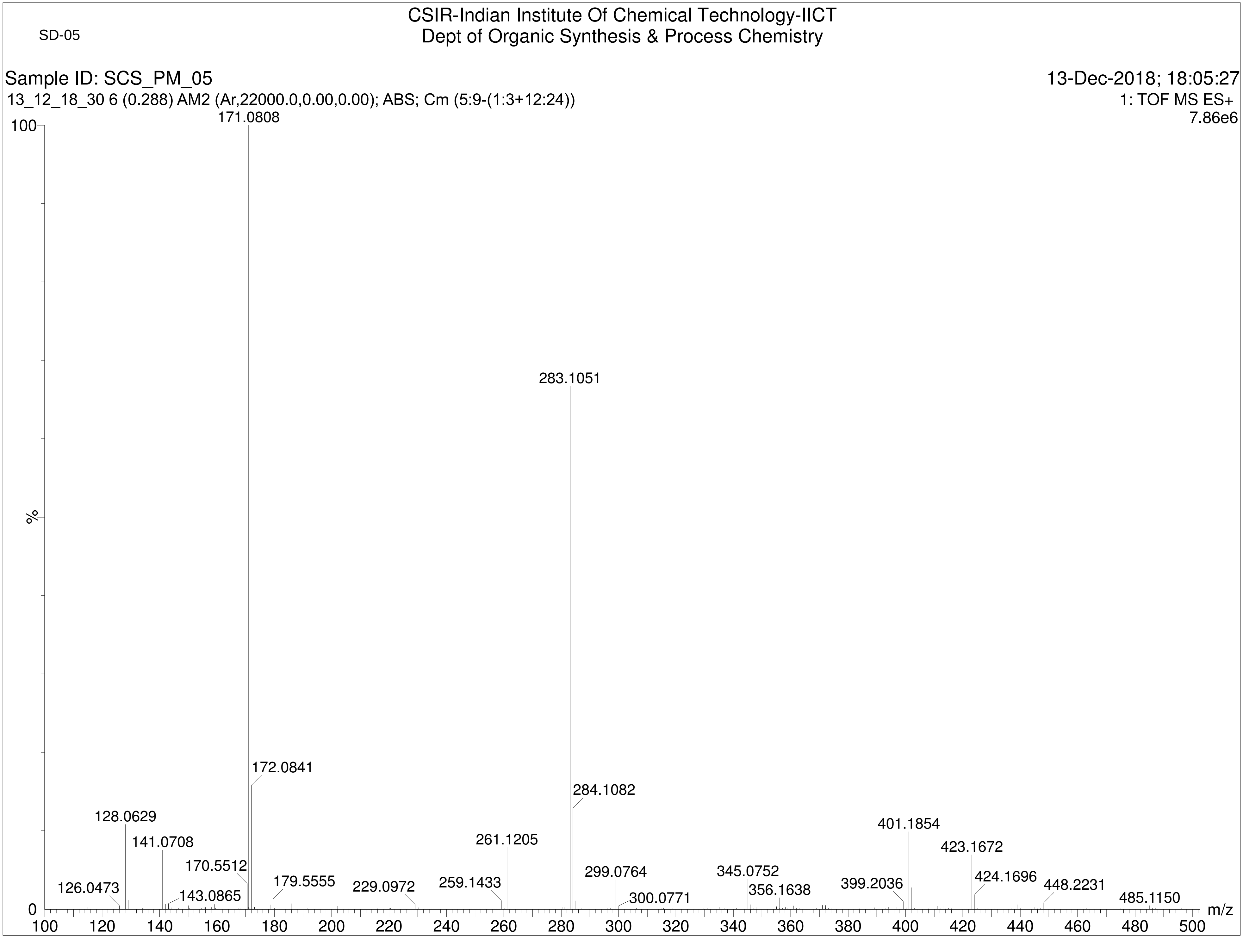

Figure S8e. Mass spectra of compound SD-05

References S

57. Wagner, F.F., *et al*. Kinetic and structural insights into the binding of histone deacetylase 1 and 2 (HDAC1, 2) inhibitors. *Bioorg.Med.Chem*. **24**, 4008-4015, doi: 10.1016/j.bmc.2016.06.040 (2016)

58. Lauffer, B. E., *et al*. Histone Deacetylase (HDAC) Inhibitor Kinetic Rate Constants Correlate with Cellular Histone Acetylation but Not Transcription and Cell Viability. *J.Biol.Chem*. **288**, 26926-26943, doi: 10.1074/jbc.M113.490706 (2013)

59. Bottomley, M.J. *et al*. Structural and Functional Analysis of the Human Hdac4 Catalytic Domain Reveals a Regulatory Zinc-Binding Domain. *J.Biol.Chem*. **283**, 26694, DOI: 10.1074/jbc.M803514200, (2008)

60. Porter, N.J., Mahendran, A., Breslow, R., Christianson, D.W.Unusual zinc-binding mode of HDAC6-selective hydroxamate inhibitors. *Proc. Natl. Acad. Sci.* U.S.A. **114,** 13459-13464, DOI: 10.1073/pnas.1718823114 (2017)

61. Vogerl, K., *et al*. Synthesis and Biological Investigation of Phenothiazine-Based Benzhydroxamic Acids as Selective Histone Deacetylase 6 Inhibitors. *J.Med.Chem*. **62,** 1138-1166, DOI: 10.1021/acs.jmedchem.8b01090 (2019)

62. Tabackman, A. A., Frankson, R., Marsan, E. S., Perry, K., Cole, K. E. Structure of 'linkerless' hydroxamic acid inhibitor-HDAC8 complex confirms the formation of an isoform-specific subpocket. *J.Struct.Biol*., **195**: 373-378, DOI: 10.1016/j.jsb.2016.06.023 (2016)

63. Somoza, J.R., *et al*. Structural Snapshots of Human HDAC8 Provide Insights into the Class I Histone Deacetylases. *Structure*, **12**, 1325-1334, DOI: 10.1016/j.str.2004.04.012 (2004)
